# Virus-specific impacts on honey bee flight performance are mediated by the octopamine pathway

**DOI:** 10.7554/eLife.111072

**Published:** 2026-11-09

**Authors:** Naomi G Kaku, Michelle L Flenniken

**Affiliations:** 1 Department of Plant Sciences and Plant Pathology, Montana State University Bozeman United States; 2 Pollinator Health Center, Montana State University Bozeman United States; The University of Texas Southwestern Medical Center United States; The University of Texas Southwestern Medical Center United States

**Keywords:** *Apis mellifera*, honey bee, deformed wing virus, sacbrood virus, flight, octopamine, Other

## Abstract

High annual honey bee colony losses are associated with environmental and biological stressors, including virus infections. In insects, the octopamine (OA) pathway orchestrates the ‘fight-or-flight’ response, regulating energy mobilization, temperature, and flight. We determined that sacbrood virus (SBV) infections induce expression of an OA receptor and enhance honey flight performance, whereas deformed wing virus (DWV) infections reduce flight performance, but how viruses interface with this pathway remained unknown. To elucidate the relationships between the OA response, virus infection, and flight, honey bees were infected with SBV or DWV and exposed to OA, epinastine (EP) – an OA receptor antagonist, or both OA and EP; flight and gene expression were assessed. Pharmacologic manipulation revealed that OA supplementation rescued flight deficits in DWV-infected bees, but diminished performance in SBV-infected bees, while blocking OA receptors altered these effects. Transcriptome analyses indicated that SBV infections, and DWV infection with OA treatment, activated honey bee metabolic pathways, and that SBV-infected bees had greater expression of genes involved in OA synthesis, unless treated with OA. These results provide mechanistic insight for virus-specific impacts on honey bee flight, which may have consequences for foraging efficiency, colony health, and virus transmission.

## eLife digest

Honey bees are important pollinators of plants, including those that produce fruit, nut, and vegetable crops. They live in colonies of about 30,000 individuals, most of which are managed by beekeepers. Since 2008, honeybee colony deaths have been high; in the USA, these losses averaged 40% annually from 2008 to 2025. Beekeepers can divide one colony into two to help offset those losses, which helped maintain the overall US honey bee colony population at around 3 million colonies.

Many factors are associated with poor honey bee colony health and/or death, including lack of available forage (i.e., flowers from which bees collect nectar and pollen), chemical exposure, mites, and pathogens – including viruses. Honey bees are commonly infected with several viruses, and adults may harbor high levels of deformed wing virus and sacbrood virus, despite lacking morphological symptoms of these viruses (i.e., wing deformity and larval death, respectively).

Since flight requires a lot of energy, Kaku and Flenniken thought that virus-infected bees may have less energy, and that flight performance may be a suitable quantitative metric of bee health. To investigate the impact of asymptomatic virus infections on honey bees, these scientists measured the flight abilities of honey bees using a flight mill. The flight mill works similarly to a treadmill and enabled the researchers to measure how far and fast individuals flew after they were infected with viruses in the laboratory.

Deformed wing virus affected bees as they expected: they flew shorter distances at slower speeds compared to uninfected bees. Surprisingly, though, honey bees infected with sacbrood virus flew farther distances at faster speeds than uninfected bees. Their previous research showed that honey bees infected with sacbrood virus expressed more of the octopamine ß receptor 2, a cellular receptor that binds octopamine, a small molecule comparable to the “fight or flight” stimulating molecule in mammals called norepinephrine. Kaku and Flenniken confirmed the involvement of this pathway in the flight performance of virus-infected bees through pharmacological manipulation, flight performance assays, and analysis of associated gene expression.

Adding octopamine improved flight performance in bees infected with deformed wing virus but worsened flight performance in bees infected with sacbrood virus. Blocking octopamine receptors changed these effects. Gene expression analyses showed that the two viruses affected metabolism and octopamine production differently. These findings suggest that different viruses perturb octopamine signalling pathways in distinct manners, which in turn impact flight performance, potentially affecting foraging, colony health and virus spread.

Moreover, the results of Kaku and Flenniken demonstrate virus-specific impacts on honey bee flight, despite the lack of visible symptoms. Seemingly asymptomatic honey bees infected with deformed wing virus may not be able to fly as far from the colony to obtain food for the colony, whereas sacbrood virus-infected bees may be able to travel farther – though co-infections will neutralize those effects. Since octopamine alleviates the flight impairment of deformed wing virus-associated bees, it will be important to further investigate the pathways that govern the effects of viruses on honey bees. In the future, better understanding of virus-host interactions may lead to ways to reduce virus-associated honeybee colony deaths.

## Introduction

Honey bees (*Apis mellifera*) are eusocial, generalist pollinators that live in densely populated colonies of ~30,000 individuals (Farrar, 1937; Chabert et al., 2021; Winston, 1991). The survival of each colony depends on the coordinated activities of thousands of individual bees that provision nutritional resources, rear brood, and regulate internal hive temperature (Winston, 1991; Simpson, 1961; Jones and Oldroyd, 2006; Kleinhenz et al., 2003). Colony losses in the U.S. averaged 40% from 2008 to 2025 (Bruckner et al., 2023; Traynor et al., 2016; Seitz et al., 2015; vanEngelsdorp et al., 2010; Nearman et al., 2025); these losses are associated with several abiotic and biotic stressors, including parasites and pathogens (Genersch et al., 2010; Genersch and Aubert, 2010; Goulson et al., 2015; Nazzi et al., 2012; Kane and Faux, 2026). Honey bee pathogens include bacteria, microsporidia, and viruses (Grozinger and Flenniken, 2019; Tarpy and Keller, 2021). Viruses may be transmitted vertically, horizontally, or by vectors, including the *Varroa destructor* mite (de Miranda et al., 2013; Chen et al., 2006; Amiri et al., 2019; Amiri et al., 2016; Fievet et al., 2006). *Varroa* mites feed on honey bees, and infestations may kill colonies (Nazzi and Le Conte, 2016). In addition, *Varroa* mites vector viruses, including deformed wing virus (DWV, *Iflavirus aladeformis*), which replicates in the mite, and others that are mechanically transmitted, such as sacbrood virus (SBV, *Iflavirus sacbroodi*) (Damayo et al., 2023; Lanzi et al., 2006; Ghosh et al., 1999). Most characterized bee-infecting viruses have positive single-stranded RNA (+ssRNA) genomes, including members of *Iflaviridae* (e.g. DWV and SBV). Virus infections in honey bees may remain seemingly asymptomatic, cause morphological symptoms, paralysis, and/or result in death (Grozinger and Flenniken, 2019; Tentcheva et al., 2004; Gauthier et al., 2007; Chen et al., 2014; McMenamin and Flenniken, 2018; Chen and Siede, 2007). Honey bees that lack morphological symptoms may still harbor virus infections that impact lifespan and flight performance (Traynor et al., 2016; Kaku et al., 2025; Wells et al., 2016; Osabutey et al., 2025; Benaets et al., 2017; Faurot-Daniels et al., 2020; D’Alvise et al., 2019; Ferreira et al., 2025).

Flight is an energetically taxing behavior that is essential for honey bee foraging, defecation, and mating (Heidinger et al., 2014; Floris et al., 2021; Gmeinbauer and Crailsheim, 1993; Klein et al., 2019; Drummond, 2022; Graham and Langstroth, 1992), and flight muscles are essential for temperature regulation (i.e. fanning to cool and ‘shivering’ for heat; de Miranda et al., 2013; Brodschneider et al., 2022; Kaya-Zeeb et al., 2022b; Stabentheiner et al., 2012; Stabentheiner et al., 2010). In our previous work, we used flight as a proxy for overall bee health and demonstrated that honey bees harboring high levels of DWV without obvious symptoms had impaired flight performance (Kaku et al., 2025). Unexpectedly, we determined that the flight performance of SBV-infected honey bees was enhanced and linked to the metabolism-stimulating, stress-induced octopamine (OA) pathway (Kaku et al., 2025). Abiotic and biotic stressors may induce enhanced activity of stress pathways, including the OA response. Invertebrates synthesize OA, a ‘fight-or-flight’ neurohormone analogous to norepinephrine, which facilitates energy production for taxing activities including flight (Li et al., 2016; Schulz and Robinson, 1999; Fussnecker et al., 2006; Blenau et al., 2020; Adamo, 2008). The OA response may also be triggered by other stressors, including pathogen infections (Kaku et al., 2025; Gage et al., 2018; Mayack et al., 2015).

**Figure 1.**
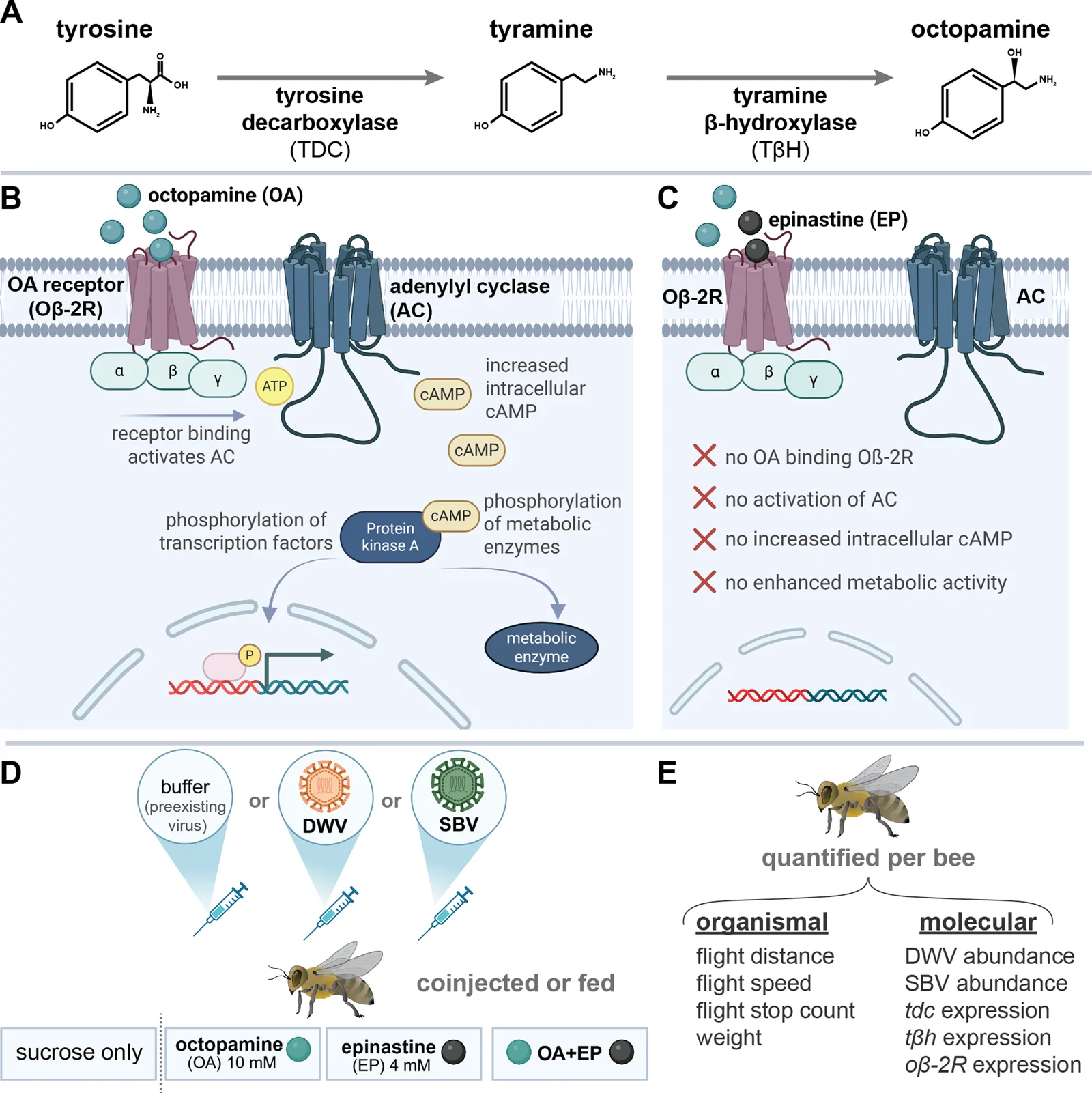
Octopamine (OA) signaling pathway (i.e. synthesis, activation, inhibition and experimental design). (**A**) OA is synthesized by conversion of tyrosine to tyramine by tyrosine decarboxylase (TDC) and tyramine conversion to OA by tyramine β-hydroxylase (TβH). (**B**) OA β–2 receptor (Oβ–2R) is one of the most abundant and specific OA-detecting receptors. Upon activation of Oβ–2R, a G-coupled protein receptor, adenylyl cyclase (AC) is activated and increases intracellular cyclic adenylyl monophosphate (cAMP) levels. High cAMP levels induce protein kinase A activity, which phosphorylates metabolic enzymes and transcription factors that enhance metabolic activity. (**C**) An OA receptor antagonist, epinastine (EP), inhibits OA binding and prevents activation of the Oβ–2R pathway. (**D**) To evaluate relationships between virus infection and the OA response, honey bees were inoculated with either deformed wing virus (DWV) or sacbrood virus (SBV) via injection or mock infected. Bees were exposed to either OA, EP, or both OA and EP via co-injection or feeding; all bees were fed sucrose. (**E**) Impacts of experimental conditions were quantified in individual bees 72 hrs post-infection. Panel B and C created with BioRender.com.

Whether OA signaling is immunosuppressive or immunoenhancing is dependent on multiple factors, and its role in honey bee antiviral defense has not been thoroughly investigated (Huang et al., 2012; Liu et al., 2019). Honey bee behaviors may be influenced by multiple monoamine neurohormones including OA, serotonin, dopamine, and tyramine (Schulz and Robinson, 1999; Barron et al., 2007; White et al., 2021; Schulz et al., 2004; Patil and Joshi, 2025; Linn et al., 2020; Cook et al., 2017). OA promotes movement, neuromuscular signaling, and metabolism. To synthesize OA, tyrosine is first converted to tyramine by tyrosine decarboxylase (TDC); tyramine is the precursor neurohormone to OA and is generally associated with reduced flight and movement (Fussnecker et al., 2006; Brembs et al., 2007; Saraswati et al., 2004). Tyramine may serve as an antagonistic modulator of behavior to OA, or tyramine β-hydroxylase (TβH) may convert it to OA (Figure 1A). One of the most abundant OA receptors in honey bees is a G-coupled protein receptor, Oβ–2R (Kaya-Zeeb et al., 2022a). When OA binds to Oβ–2R, it activates adenylyl cyclase (AC), which increases intracellular concentration of cyclic adenylyl monophosphate (cAMP). Elevated cAMP activates cAMP-dependent protein kinase A (PKA), which phosphorylates metabolic enzymes and transcription factors that generally enhance metabolic activity (Figure 1B; Kaya-Zeeb et al., 2022b; Blenau et al., 2020; Kaya-Zeeb et al., 2022a; Sassone-Corsi, 2012).

We hypothesized that SBV infections, which correlate with higher expression of an OA receptor (Oβ–2R; Kaku et al., 2025), activate the OA response, which results in enhanced flight performance. Therefore, we predicted that OA treatment would enhance honey bee flight performance regardless of virus infection. Epinastine (EP) is a highly specific OA receptor antagonist, exhibiting at least four orders of magnitude greater affinity for OA receptors than for other honey bee biogenic amine receptors (Roeder et al., 1998; Degen et al., 2000). Therefore, we hypothesized that EP treatment would reduce honey bee flight performance and negate SBV-associated enhanced flight performance.

## Results

To test these hypotheses, we exposed virus-infected honey bees to OA, EP, or both OA and EP and assessed flight performance and gene expression (e.g. *tdc*, *tβh*, and *oβ–2R*) in three independent experiments (i.e. exp-1, 2, and 3; Figure 1 and Source data 1, data S1). Specifically, age-matched, one-day-old adult bees (n=336) were experimentally infected with DWV (n=117), SBV (n=75), or mock infected (buffer injection, n=144) and treated with OA (10 mM), EP (4 mM), or both OA and EP via injection or feeding, or not treated; all bees were fed 50% sucrose syrup ad libitum. Treatments of OA and EP were given in 2 µL injections or fed at the same concentration in sucrose syrup; doses were in-line with previous studies (Kaku et al., 2025; Kaya-Zeeb et al., 2022b; Fussnecker et al., 2006; Roeder et al., 1998). Exposure to OA and/or EP via injection was temporally limited, whereas ad libitum feeding provided continuous exposure (Figure 1).

### Quantification of experimentally introduced and naturally occurring honey bee virus infections

Honey bees used in this study were sourced from managed colonies that are naturally exposed to pathogens. To limit variation associated with confounding infections, bees were collected upon emergence under laboratory conditions (de Miranda et al., 2013; Kaku et al., 2025; Carrillo-Tripp et al., 2016; Brutscher et al., 2017). Therefore, in addition to the use of quantitative polymerase chain reactions (qPCR) to determine the abundance of DWV and SBV in individual honey bee samples, PCR was used to test for potential preexisting infections and detected pathogens were quantified using qPCR, as described previously (Kaku et al., 2025; Source data 1, data S1-S3). We determined that honey bees in exp-1 had preexisting DWV infections and bees in exp-2 and exp-3 had preexisting infections of DWV and SBV (Appendix 1—figure 1). Most bees analyzed herein had either experimentally introduced or preexisting DWV infections (n=325/336) and 40% had experimentally introduced or preexisting SBV infections (n=134/336). DWV infections ranged between 1 × 10^3^ and 7.1 × 10^10^ copies per 2 µg of RNA (~7 × 10^3^ – 8 × 10^11^ DWV copies per bee). SBV infections were between 1.2 × 10^3^ and 1.2 × 10^10^ copies per 2 µg of RNA (~8.3 × 10^4^ – 8.3 × 10^11^ SBV copies per bee). Due to high DWV and SBV prevalence, there were no virus-free honey bees. Mock-infected bees harbored low virus levels (i.e. an average of 3 × 10^5^ DWV copies and 2 × 10^3^ SBV copies per 2 µg RNA) and inoculation resulted in higher virus loads (i.e. an average of 2 × 10^8^ DWV copies and 2 × 10^8^ SBV copies per 2 µg RNA), p=2 × 10^–16^ and 5 × 10^–11^, respectively. To compare the impact of varying virus levels and treatments, we evaluated several flight metrics (i.e. flight distance, duration, speed, and stop count; Source data 1, data S1) using linear mixed effect models (LMMs; complete information for each model and model selections in methods and Source data 1, data S4).

### OA and EP altered flight performance of virus infected bees

**Figure 2.**
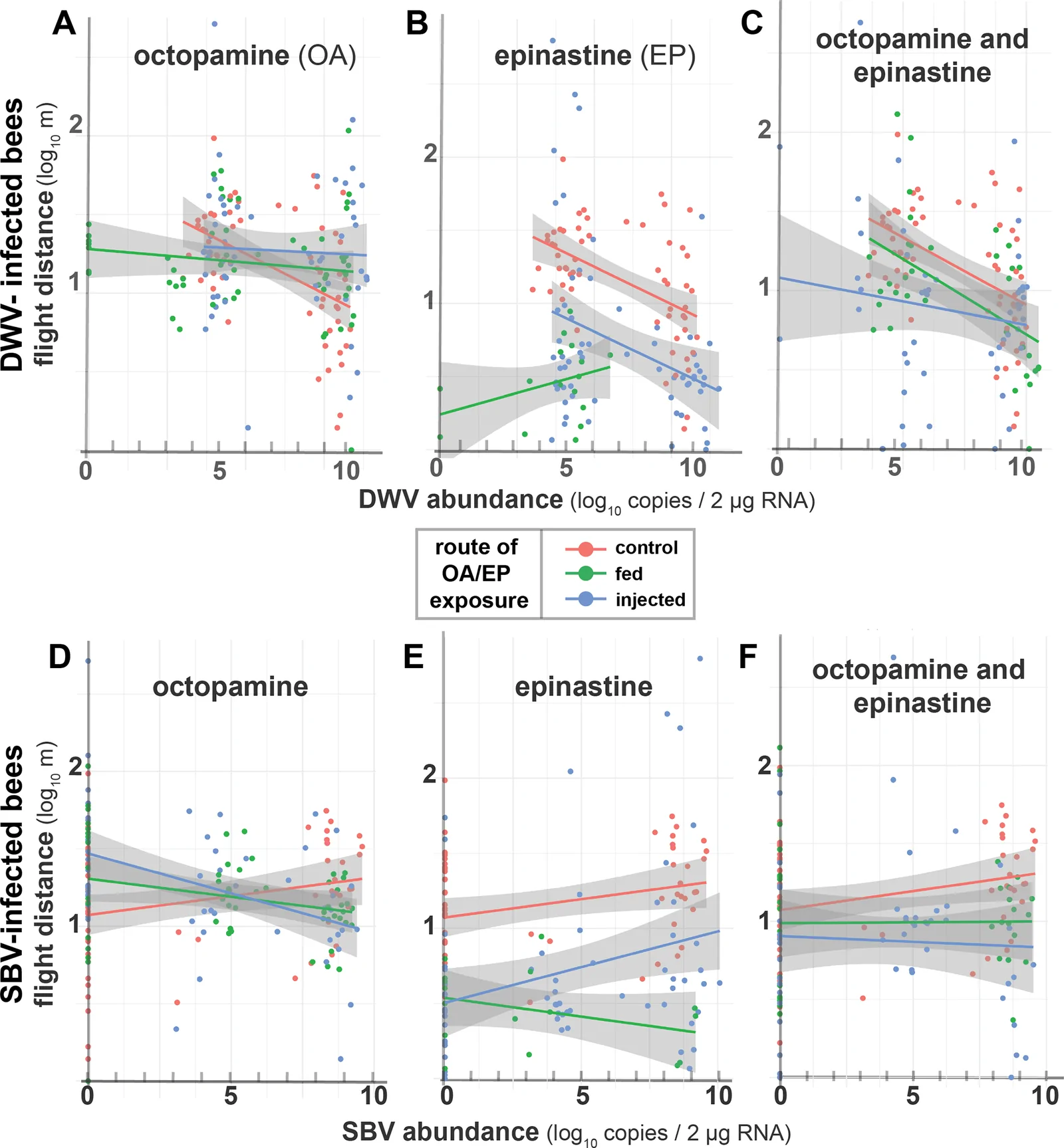
The flight performance of honey bees infected with DWV and/or SBV is differentially impacted by OA and EP treatment. To evaluate the involvement of the OA pathway on virus infection and/or flight performance, virus- or mock-infected honey bees were exposed to OA, EP, or both OA and EP via co-injection (blue) or feeding (green). Honey bees were experimentally infected with or had preexisting infections of DWV (**A–C**) or SBV (**D–F**) in three experiments. Flight distance (log_10_ m) was evaluated using linear mixed effect models to quantify impacts of DWV and/or SBV infection in the context of OA and/or EP treatment. Each data point represents an individual bee (n=336); colors indicate treatment (i.e. bees fed only sucrose in pink, bees fed OA and/or EP in green, and bees injected with OA and/or EP in blue) and gray areas represent 95% confidence intervals. Linear mixed models with estimates in Source data 1, data S4. (**A**) In bees fed only sucrose, DWV infections negatively impacted flight distance (p=0.005), whereas DWV-infected bees treated with OA flew further distances (i.e. average flight distance was similar to mock-infected bees fed sucrose, p=0.61 and 0.08, respectively). (**B**) DWV-infected bees injected with EP flew shorter distances than DWV-infected bees fed only sucrose (p=0.002). (**C**) Co-injection of OA and EP negatively impacted the distance DWV-infected honey bees flew (p=0.020). (**D**) OA treatment did not enhance flight performance of SBV-infected bees (p=0.69 and 0.60). (**E**) Honey bees with SBV infections fed or injected with EP flew shorter distances than SBV-infected bees fed only sucrose (p=0.022 and 0.004, respectively). (**F**) Bees with SBV infections fed or injected with OA and EP resulted in similar flight distances regardless of SBV infection level.

The previously described DWV-associated flight impairment relative to mock-infected bees (Kaku et al., 2025), was recapitulated in this study (i.e. DWV-infected bees flew shorter distances [p=0.005]). Mock-infected bees from this sample cohort, which harbored low virus levels, flew an estimated 45 m, whereas bees harboring high DWV levels (i.e. >10^8^ DWV RNA copies/2 µg RNA) flew only ~9 m. In line with our hypothesis, DWV-associated flight impairment was alleviated by OA feeding or injection (i.e. average flight distance was similar to mock-infected bees fed sucrose, p=0.61 and 0.08*,* respectively; Figure 2A). Honey bees that received EP flew shorter distances (p=0.002), but the effect of feeding EP was not assessable since bees with experimentally introduced DWV infections that were fed EP died, and there were only 14 bees with low DWV infection levels (Figure 2B, Source data 1, data S4). Co-injection of OA and EP negatively impacted the distance DWV-infected honey bees flew (p=0.020; Figure 2C). Counter to our hypothesis, OA-treatment did not enhance flight performance of SBV-infected bees (p=0.69 and 0.60; Figure 2D, Source data 1, data S4). SBV-infected bees that were treated with EP or co-treated with both EP and OA via injection flew shorter distances than untreated SBV-infected bees (p=0.022 and 0.004, respectively), whereas feeding had no impact (Figure 2E–F).

The amount of time a honey bee was actively flying was also assessed as an indicator of energetic capacity (Appendix 1—figure 2). Honey bees harboring high DWV levels (i.e. >10^8^ DWV copies/2 µg RNA) flew only 14 min (p=0.022), relative to mock-infected bees with low virus infection levels (average of 3 × 10^5^ DWV copies/2 µg RNA), which flew for 26 min. Honey bees with high SBV levels flew for similar durations to mock-infected bees (i.e. 28 min, p=0.58), which is consistent with previous results (Kaku et al., 2025). In line with our hypotheses, mock-infected bees with low virus infection levels flew longer when they were fed OA (i.e. 49 min; p=0.0001). DWV-associated flight impairment was mitigated in honey bees that were supplemented with OA, which flew for similar durations to mock-infected bees (i.e. 28 min, p=0.76). However, counter to our hypothesis, when SBV-infected bees were injected or fed OA, flight duration was significantly reduced (i.e. 18 and 12 min, respectively; p=0.04 and 0.0009).

To quantify the impact of virus and OA and/or EP treatment on honey bee flight speed, we compared average and peak speeds. Average flight speed (i.e. total distance/total flight duration) was similar across all conditions except in bees fed EP, which flew at slower average speeds (p=3 × 10^–4^; Source data 1, data S4). We also compared peak speed (i.e. maximum speed reached during flight sustained >10 s; Appendix 1—figure 2). Similar to previously reported results (Kaku et al., 2025), DWV-infected bees had slower peak speeds than mock-infected bees (p=0.016). SBV-infected bees flew similar speeds to mock-infected bees (p=0.07), but peak speeds were reduced when SBV-infected bees were fed or injected with OA (p=0.004, p=0.0006; Source data 1, data S4). Mock-infected bees flew at peak speeds of ~3.3 km/hr, honey bees with high levels of SBV (i.e. 10^8^ SBV copies/2 µg RNA) flew ~3.8 km/hr, whereas highly SBV-infected bees injected or fed OA flew an estimated 2.0 or 2.5 km/hr, respectively (p=0.001 and 0.022).

In addition to flight metrics, post-flight thorax temperatures were measured within a subset of individual bees (n=130) since flight induces heat and the OA response is involved in honey bee temperature regulation (Kaya-Zeeb et al., 2022b; Kaya-Zeeb et al., 2022a; Source data 1, data S1 and S4). The ambient temperature for all flight assays was 22 °C, and post-flight thorax temperatures in honey bees maintained in control conditions averaged 28 °C. DWV-infected bees had similar post-flight temperatures, and highly SBV-infected bees were 2 °C cooler than control (p=0.04). Honey bees with DWV infections that were fed OA had thorax temperatures that were 2.5 °C higher than bees not fed OA. Whereas the thorax temperatures of bees with DWV infections were 5.6 °C lower in bees co-fed OA and EP (p=0.027 and 1 × 10^–6^). Likewise, honey bees with high SBV levels that were injected with OA also had reduced thorax temperatures post-flight (i.e. ~3 °C lower) (p=0.03; Source data 1, data S4).

### Virus-specific outcomes of experimentally manipulated octopamine pathway explained by differential expression of key genes

**Figure 3.**
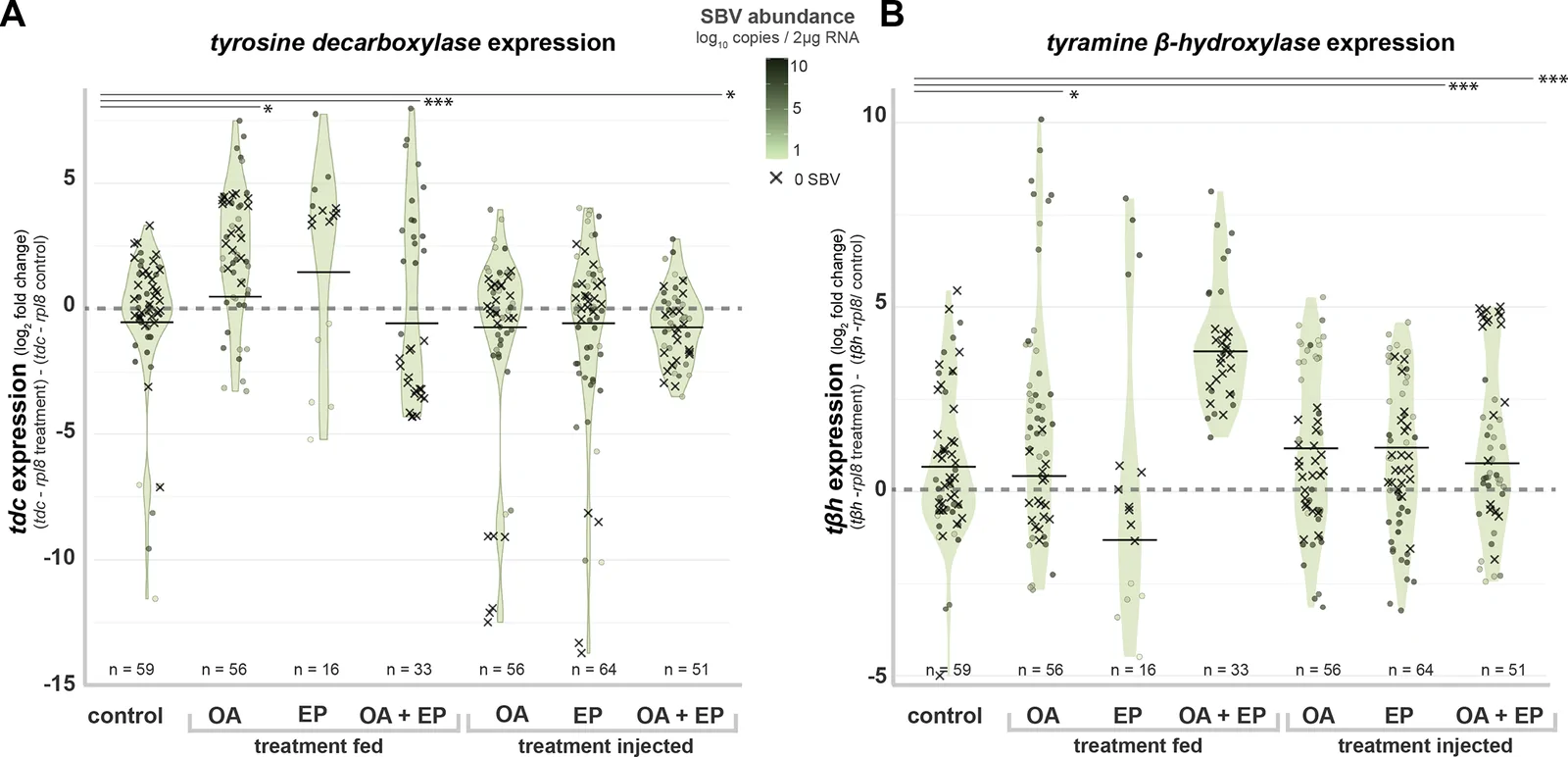
Expression of *tyrosine decarboxylase* (*tdc*) and *tyramine β-hydroxylase* (*tβh*) across treatments and SBV infection. The relationships between honey bee *tdc* and *tβh* expression in the context of virus infection and treatments were evaluated using a linear mixed effect model that included SBV abundance and OA/EP treatments as fixed effects with an interaction. Individual honey bee data plotted by treatment group with either (**A**) *tdc* expression or (**B**) *tβh* expression. Gene expression was calculated as log_2_ fold change using the ΔΔCt method relative to housekeeping *rpl8* and compared to mock-infected bees. SBV abundance was calculated as log_10_ copies/2 µg RNA. The background violin plots represent the 95% confidence interval, and the horizontal line in each violin represents the median. SBV abundance is represented by a green color scale and SBV-negative samples with an ‘X’. When SBV abundance did not impact *tdc* or *tβh* expression the dark green points are evenly distributed above and below the control ‘0’ fold change line, whereas unequal distribution indicates an SBV-specific effect; * p<0.05, **p<0.01, and ***p<0.001. (**A**) Positive association between SBV abundance and *tdc* expression (p=0.021). SBV-infected bees fed OA or both OA and EP had greater *tdc* expression levels than uninfected bees fed only sucrose (p=0.03 and 2x10^–10^). (**B**) Expression of *tβh* was greater in SBV-infected bees than uninfected bees (p=0.022). SBV-infected bees fed OA had greater *tβh* expression levels than uninfected bees fed sucrose (p=2 × 10^–5^). SBV-infected bees injected with EP or co-injected with OA and EP had lower *tβh* expression (p=2 × 10^–5^ and 4x10^–9^).

**Figure 4.**
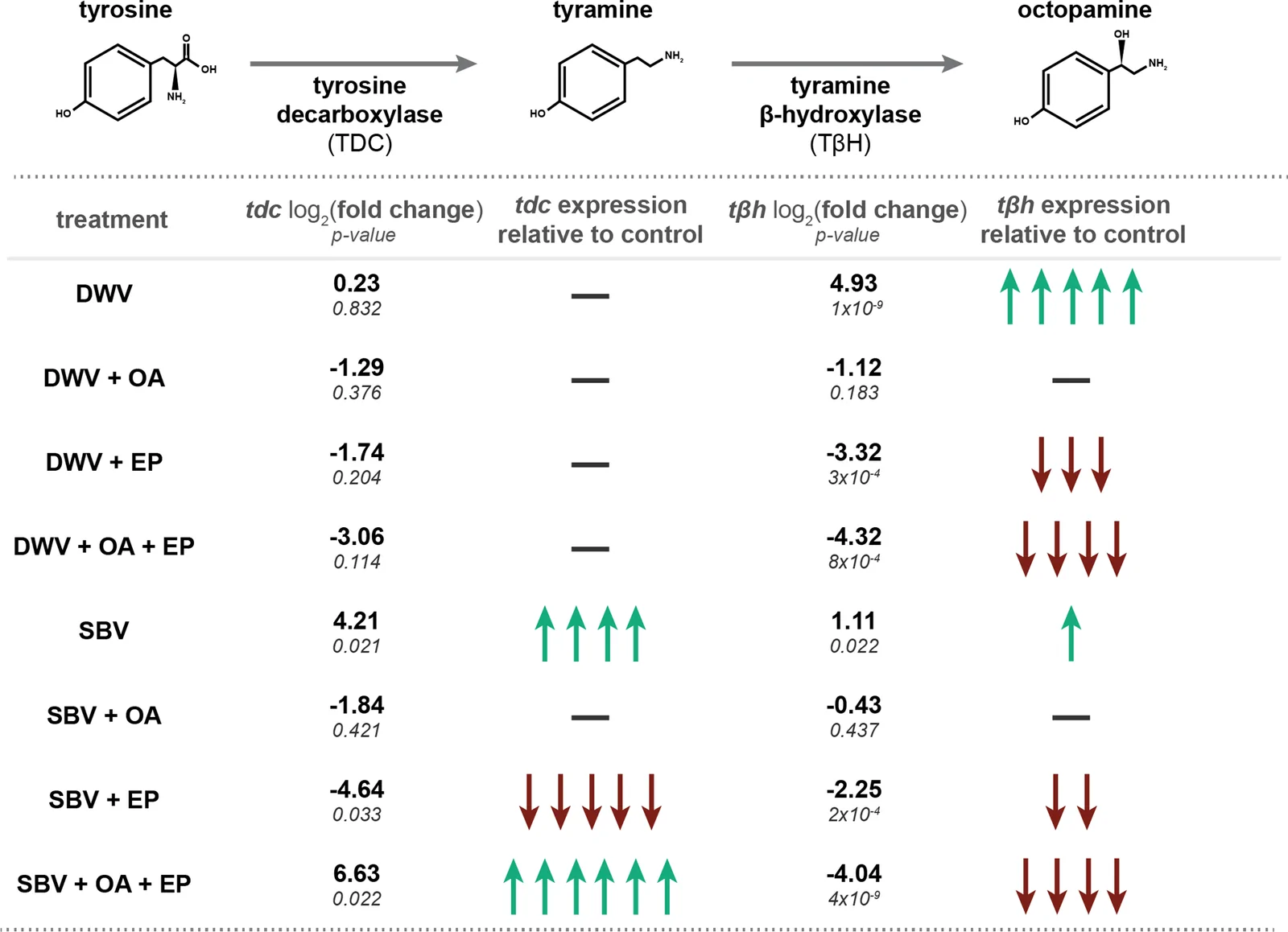
Relative *tdc* and *tβh* expression in honey bees with high DWV or SBV levels. (**A**) Tyrosine is converted via tyrosine decarboxylase (TDC) to tyramine. Tyramine is a neurohormone that acts as a behavioral antagonist to octopamine (OA), resulting in reduced movement in invertebrates. Tyramine may act as a signaling neurohormone, or tyramine β-hydroxylase (TβH) may convert it to OA. (**B**) A linear mixed model was used to assess the relative expression of *tdc* and *tβh* in honey bees harboring high levels of either deformed wing virus (DWV) or sacbrood virus (SBV; i.e. 10^8^ virus copies/2 µg RNA) in the context of OA and/or epinastine (EP) injection. Estimates of relative *tdc* and *tβh* expression were generated based on data from 336 honey bees. The single bold dash indicates *tdc* or *tβh* expression was similar in virus-infected bees and mock-infected bees, and arrows indicate estimates that were higher or lower than controls where each arrow represents fold change of 1; Source data 1, data S2.

Induction of the OA pathway was previously implicated in the enhanced flight performance of SBV-infected honey bees, which had elevated expression of *oβ–2R* (Kaku et al., 2025). When OA binds to Oβ–2R, it activates AC, increases cAMP concentration, thereby activating cAMP-dependent PKA, which phosphorylates enzymes and transcription factors that enhance metabolic activity (Figure 1B; Kaya-Zeeb et al., 2022b; Blenau et al., 2020; Kaya-Zeeb et al., 2022a; Sassone-Corsi, 2012). To determine if DWV, SBV, or different experimental treatments were associated with differences in OA synthesis, we quantified the expression of *tdc* and *tβh* relative to mock-infected honey bees fed only sucrose (Source data 1, data S1, Figure 3). We evaluated the relationship between *tdc* expression and multiple factors including DWV abundance, SBV abundance, and experimental treatments (i.e. OA and/or EP, fed/injection) using LMMs which explained 91% of the data variation (Source data 1, data S4). SBV infection was associated with increased *tdc* expression (p=0.021), and SBV-infected bees fed OA or co-fed OA and EP also expressed higher *tdc* levels (p=0.030 and 2 × 10^–10^, respectively). Expression of *tdc* was lower in SBV-infected bees co-injected with EP, and SBV and DWV-coinfected bees that were fed OA or both OA and EP (p-values = 0.033, 0.022, 0.006, 0.004, respectively; Figure 3A, Source data 1, data S4). Expression of *tdc* was not associated with DWV infection, or DWV infection with OA and/or EP treatment (p-values all >0.2, Source data 1, data S4, Appendix 1—figure 3). We also examined the effects of virus infection in the context of experimental OA and EP treatments on *tβh* expression (i.e. in bees with detectable *tβh* levels n=306/336; Figure 3B). An LMM that included DWV abundance, SBV abundance, treatment, and *tdc* expression as explanatory variables explained 85% of the variation in *tβh* expression (Source data 1, data S4). Expression of *tdc* and *tβh* were positively correlated (with a *tdc:tβh* ratio of 1:1.6, p=0.004; Appendix 1—figure 4). Expression of *tβh* was higher in SBV and DWV infected bees (p=0.022 and 1x10^–9^, respectively), suggesting more tyramine is converted to OA during DWV or SBV infection (Figure 3B). Results from these models indicate that only SBV infections were associated with greater expression of both *tdc* and *tβh* relative to mock-infected bees with low preexisting virus levels, whereas SBV-infected honey bees that were co-injected with OA expressed similar levels of *tdc* or *tβh* as mock-infected bees (p=0.421 and 0.437, respectively). This result suggests that the elevated expression of genes involved in OA synthesis in SBV-infected honey bees is reduced by OA supplementation and partially explains the impaired flight performance of these bees relative to untreated SBV-infected honey bees (Figure 4). For OA to stimulate metabolic activity and energy production, OA must activate receptors including Oβ–2R (Kaya-Zeeb et al., 2022a). To identify variables that contribute to the range of *oβ–2R* expression, we compared LMMs including *tβh* expression, *tdc* expression, SBV abundance, and OA/EP treatment, which explained 95% of the data variation (Appendix 1—figure 4). We considered including DWV abundance as a fixed effect in this model, but it did not appreciably contribute to *oβ–2R* expression (Source data 1, data S4). These results corroborate our previous findings that SBV, but not DWV, was associated with increased *oβ–2R* expression (Kaku et al., 2025). The strongest predictors of *oβ–2R* expression levels were *tdc* and *tβh* expression (p=4.4 × 10^–5^ and 3.9 × 10^–5^) and SBV was positively associated with *tdc* and *tβh* expression (p=0.0006; Source data 1, data S4).

### Transcriptome level impacts of virus infection, flight, and OA treatment

We hypothesized that flight-impacting virus infections would result in differential expression of genes involved in stress, metabolic, and immune pathways. To test this, the transcriptomes of SBV, DWV, DWV and OA, or mock-infected honey bees were compared. Honey bees were obtained from a previous experiment and included individuals that did or did not fly (Kaku et al., 2025). RNA was prepared for high-throughput sequencing on an Illumina NovaSeq X Plus 25B (n=36 total individual bee samples, 3–5 per treatment group; Source data 1, data S6). An average of 25 million reads was obtained for each sequencing library and mapped to the *A. mellifera* assembly HAv3.1 (Weinstock et al., 2006; Elsik et al., 2014). To evaluate similarities and differences between the transcriptional responses of honey bees with flight-impacting virus infections, we compared DWV, SBV, and DWV and OA injected bees relative to mock infected bees immediately post-flight since these bees had little to no detectable preexisting infections, whereas the no-flight mock infected bees had preexisting DWV infections (Source data 1, data S5 and S6).

**Figure 5.**
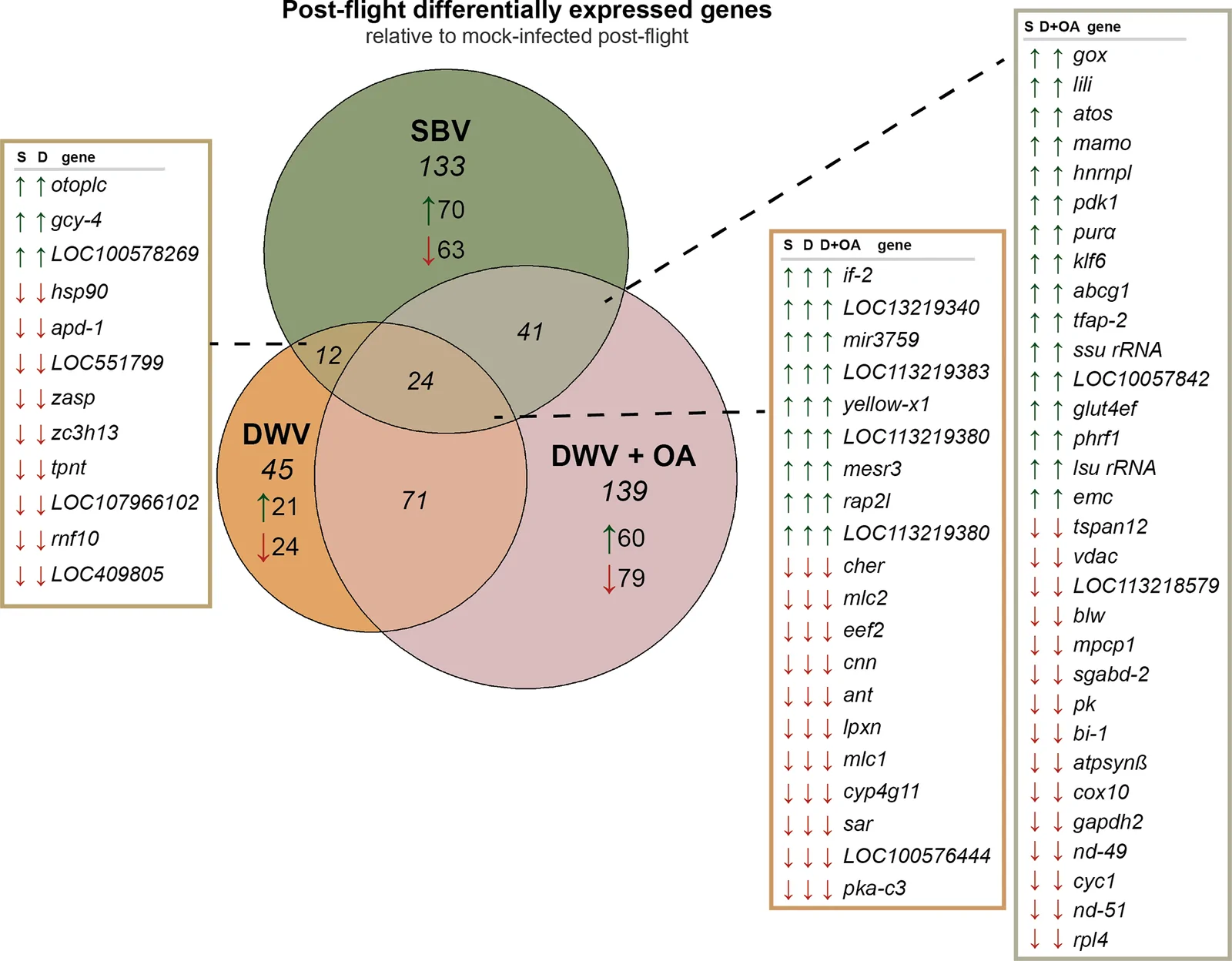
Transcriptome level comparison of honey bees that were SBV infected, DWV infected, or DWV infected and OA-treated. Gene expression was evaluated in honey bees that were mock infected, SBV infected, DWV infected, or DWV infected bees that were co-injected with OA (DWV +OA) after flight. Differentially expressed genes (DEGs) for each treatment group were identified relative to mock infected bees and compared using a Venn diagram. Full list of DEGs, fold change, and Benjamini-Hochberg corrected significance values are included in Source data 1, data S6.

Hundreds of differentially expressed genes (DEGs) were identified in SBV, DWV, and OA-treated DWV-infected bees (Figure 5). DWV-infected bees with and without OA treatment shared 95 DEGs, which was more than they shared with SBV-infected bees, indicating that there are virus specific transcriptional responses. There were 24 genes shared among SBV, DWV, and DWV and OA injected bees, including elevated expression of those involved in the Ras-Raf-MEK-ERK pathway (including *mesr3* and *rap2l*), which is associated with metabolism, muscle development, and the cAMP/PKA pathway (Dumaz and Marais, 2005). Genes with lower expression were involved in muscle function (i.e. *mlc2, mlc1, cher, sar*), energy production (i.e. *cnn, ant, pka-c3*), and an inhibitor of proteolytic activity (i.e. *cyp4g11*).

There were many genes unique to SBV and DWV infections (Figure 5). Honey bees with SBV infections exhibited lower expression of several genes associated with the citric acid cycle (*acsl6*, *cg5065*, *acyl-coA delta* (Nearman et al., 2025), *mcad*, *nd-13a*, *nd-13b*, *nc73ef*, and *mdh2*) and general muscle function and tissue repair genes (*tmp-1, tmod, plastin-2, and fgf17*). SBV-infected bees had elevated expression of *rgs11* – which increases G-protein α-subunit active state to inactive to expedite the G-coupled protein response (Snow et al., 1998), *titin* – which contracts stretched/striated muscle tissues (Wang et al., 1979), and several immune genes such as *argonaute-2*, *relish*, and *domeless* (Source data 1, data S6 and S7). DWV-infected bees exhibited greater expression of several genes associated with immune function including *protein mahjong*, *spastin*, and a small heat shock protein, *protein-lethal(2) essential for life-like* (Chabert et al., 2021; Source data 1, data S6). The DEGs unique to DWV-infected bees post-flight that exhibited lower expression included genes involved with muscle function and structure (*obscurin*, a titin homolog, *ryr*, *actin*, *mhc*, *stacl*, *tpm1*, and *zasp66*) and ATP generation (*citrate synthase, adenylate kinase 1, ADP/ATP translocase*).

Since OA supplementation mitigated DWV-associated flight impairment, we identified differences associated with OA by comparing DEGs to untreated DWV-infected honey bees post-flight. As hypothesized, DWV-infected bees that were co-injected with OA treatment had greater expression of OA pathway genes (*oβ–2R*, *adenylyl cyclase (ac*), and CREB binding proteins) and genes involved in glucose metabolism (*gale*, *gdh*, *adpgk*, *putative glucosylceramidase 4*, *gba1lp, g6pd*, *sugarless*, *pdp*, *ND-24*, *NAD kinase*, *tret1*, *1,5-anhydro-D-fructose reductase, and igfals*; Source data 1, data S8 and S9). These bees also expressed higher levels of genes induced by high cyclic guanine monophosphate (cGMP) levels (i.e. *burgundy*, *cGMP-dependent protein kinase-like*, and *cGMP-dependent protein kinase 1*) and genes in the RAS/MAPK-pathway (*rho1*, *rac1*, and *rab5*). Untreated, DWV-infected bees expressed higher levels of several immune genes (*abaecin, domeless*, *echinoid*, and *defensin-1*) and heat shock protein encoding genes (*hsp83*, *hsp90*, *hspβ–1*) relative to DWV-infected bees treated with OA (Source data 1, data S7-S9).

After flight, both SBV-infected and OA-treated DWV-infected bees expressed lower levels of several mitochondrial associated genes (*vdac, blw, atpsynβ*, *cox10*, *klf6,* and *mcmp1*) and genes involved in energy generation (*pyruvate kinase*, *gapdh2,* and 2 *ND-49,* and *ND-51*) than mock-infected bees (Figure 5). This result suggested that flight was more taxing for virus-infected bees. In addition, transcriptome level results indicated that OA treatment during DWV inoculation resulted in enhanced energy production, since these bees expressed higher levels of genes involved in energy production post-flight relative to DWV-infected bees that did not fly (Source data 1-data S10).

Since OA treatment resulted in recovered flight performance in DWV-infected bees, we compared the transcriptomes of honey bees with similar DWV-levels that were either OA-treated or untreated. The 50 genes with the greatest difference in mean expression were identified in bees that did or did not fly (i.e. 100 total possible genes; Source data 1, data S10). There were 30 shared genes associated with OA-treatment regardless of flight status. This list included *defensin-1*, *apidermin-2*, *melittin*, *odorant binding protein 3*, *chymotrypsin-1*, two α-glucosidases, *peritrophin*, two chitinase proteins, *fibrillin-2*, *muscle protein 20*, and seven uncharacterized genes. The OA-associated list also included *vegetative cell wall protein-like (gpl-1*), which exhibited higher expression in bees that flew further distances (mock, SBV, DWV, and OA), than in DWV-infected bees. Previous studies determined that *gpl-1* expression was greater in *Nosema ceranae* infected honey bees (Chang et al., 2020), and that OA signaling was also impacted (Gage et al., 2018; Mayack et al., 2015). While the bees in our study were not *Nosema* infected (Appendix 1—figure 1), we used qPCR to confirm that bees expressed higher *gpl-1* levels when injected with OA or EP (LMM, p=2 × 10^–7^ and 4 × 10^–8^, respectively); data from additional experiments (i.e. exp-1, n=61 and exp-2, n=69; Source data 1, data S1Data S1).

We also examined expression of a subset of OA-associated genes including OA receptors (i.e. *oα–1R*, *oβ–1R*, *oβ–2R*, *oβ–3R, and oβ–4R*), enzymes involved in OA synthesis (*tdc* and *tβh*), and genes associated with Oβ–2R activation (*adenylyl cyclase* (*ac*), *protein kinase A* (*pka*), and CREB-associated protein encoding genes; Source data 1, data S9 and Appendix 1—figure 5). No sample had detectable levels of *oβ–4R; oβ–1R* and *oβ–3R* were expressed at very low levels in most samples (Source data 1, data S9; Appendix 1—figure 5). Although *oα–1R* transcripts were detected in some samples, expression levels were consistently lower than those of *oβ–2R*. Expression of *tdc*, *tβh*, *oβ–1R*, *crebA*, and *creb-binding isoform X1* was generally higher in SBV-infected honey bees, and greater *tdc* and *tβh* expression was confirmed via qPCR (exp 1–3, n=336; Figure 3, Source data 1, data S1 and S4). Honey bees that were simultaneously DWV infected and treated with OA expressed higher levels of *oβ–2R*, *oα–1R*, and *creb binding isoform X2* than untreated DWV-infected bees (Appendix 1—figure 5); greater *oβ–2R* expression was also confirmed by qPCR (LMM, n=336; p=9 × 10^–4^; Source data 1, data S1- Data S1). Expression of *oa-1R* and *tyrR* was assessed via qPCR in only one experiment since expression was generally low regardless of treatment (exp-3, n=69; Source data 1, data S1). SBV-infected bees had greater expression of *oa-1R* (p=0.017), and SBV-infected bees co-injected with EP had significantly lower expression of both *oa-1R* and *tyrR* (p=0.0006 and 0.04, respectively).

## Discussion

The interactions between virus infections, host immune and stress responses, and organismal health are complex. Insects rely on OA as a neurohormone and neuromodulator to regulate physiological processes and behaviors (Li et al., 2016; Fussnecker et al., 2006; Adamo et al., 1995; Corby-Harris et al., 2020; Bisen et al., 2026), and our previous studies indicated that viruses differentially impacted honey bee OA signaling and flight performance. Therefore, we used honey bees as a model system to further investigate these interactions at organismal and transcriptional levels.

As previously described and shown herein, inapparent (or asymptomatic) DWV infections negatively impact honey bee flight performance (i.e. flight distance, duration, and peak flight speed). Evaluation of transcriptional responses to DWV infection suggests that DWV-associated flight impairment may be partially explained by lower expression of genes involved in energy production, which were elevated in OA-treated honey bees (Figure 5, Appendix 1—figure 5, Source data 1, data S7 and S8). These results are supported by previous studies that showed DWV-infected honey bees expressed lower levels of G-coupled protein receptor encoding genes, as well as other genes involved in metabolism (Tang et al., 2021; Pizzorno et al., 2021; Chen et al., 2021). Previously, we demonstrated that the flight performance of SBV-infected honey bees was enhanced and that these bees expressed more *oβ–2R* (Kaku et al., 2025). Herein, we showed that SBV-associated flight enhancement was reduced when OA signaling was inhibited by EP, an OA receptor antagonist (Figures 2 and 4). We expected that SBV-infected bees would have compounding, enhanced flight performance when treated with OA, but unexpectedly, OA supplementation negatively impacted flight performance (Figure 2). To further investigate this physiological response, we examined the expression of key genes in the OA pathway and determined that in addition *oβ–2R*, SBV-infected honey bees expressed greater levels of *tdc* and *tβh* (Source data 1, data S1). Expression of *tdc* and *tβh* was lower in SBV-infected bees that were treated with OA (Source data 1, data S1 and S4, Figures 3 and 4). Reduced expression of these genes may limit OA synthesis, OA signaling, reduce metabolic activity, and result in less available energy for flight (Li et al., 2016). Therefore, the reduced flight performance of SBV-infected bees treated with OA may be explained by a feedback loop that regulates intracellular OA concentrations, though future studies that measure OA levels are needed to validate this hypothesis.

Depending on OA concentration and specific pathogen challenge, OA may enhance or inhibit immune function in many invertebrates, though mechanistic studies have been primarily restricted to two model organisms (Huang et al., 2012; Liu et al., 2019; Sellegounder et al., 2018; Kong et al., 2018; Kuo and Cheng, 2018). In *Drosophila*, OA treatment impacts intracellular OA concentration, in a hypothesized feedback mechanism that regulates neurohormonal signaling (Patil and Joshi, 2025; El-Kholy et al., 2015; Damrau et al., 2021). In *Caenorhabditis elegans,* OA pathways are hypothesized to be constitutively active, but downregulated upon pathogen infection to enable an effective immune response while limiting immune activity in the absence of pathogen challenge (Sellegounder et al., 2018). Transcriptomic analysis of honey bees infected with a model virus (Sindbis) that were co-injected with double-stranded RNA, a virus-associated molecular pattern, had greater expression of an OA receptor (*oβ–2R*) (Brutscher et al., 2017). Results presented herein indicate that OA treatment of DWV-infected honey bees alters expression of several genes associated with immune function including *apidermin*, *defensin*, and *melittin* (McMenamin et al., 2018; Seyam et al., 2022; Mahmoud et al., 2024; Hejníková et al., 2024). Additionally, DWV-infected bees with OA treatment had reduced expression of an odorant binding protein that was previously associated with SBV infection, indicating that it may be involved in honey bee antiviral defense (Al Naggar et al., 2023). Together, these results highlight virus-specific transcriptional responses in honey bees that may impact virus dynamics and flight performance during commonly occurring coinfections (Kaku et al., 2025; Durand et al., 2023; Ryabov et al., 2016).

Multiple OA receptors have been identified in honey bee flight muscles and neural tissues including OαR1, Oβ–2R, Oβ–1R, and Oβ–3/4 R (Kaya-Zeeb et al., 2022b). OA receptors have also been detected on invertebrate immune cells (Huang et al., 2012). In addition to their involvement in flight, OA receptors are also involved in honey bee hygienic, communicative dance, and thermoregulatory behaviors (Kaya-Zeeb et al., 2022b; Barron et al., 2007; Linn et al., 2020; Kaya-Zeeb et al., 2022a; Spivak et al., 2003; Kobayashi et al., 2023). Typically, around 12 days post-emergence honey bees change from nurse bees, which care for developing brood, to pre-forager bees and undergo physiological changes required for flight (Scofield and Amdam, 2024; Johnson, 2008; Roberts and Elekonich, 2005). As honey bees age and become foragers, OA levels in the brain increase (Schulz and Robinson, 1999; Schulz et al., 2004; Kaatz et al., 1994). Honey bees infected with the microsporidian parasite, *Nosema*, have greater OA levels than uninfected bees (Gage et al., 2018; Mayack et al., 2015) and are prone to precocious foraging (Goblirsch et al., 2013), although *Nosema* levels did not appreciably impact honey bee flight capability (Wells et al., 2016). These findings are in-line with SBV-induced flight enhancement that correlated with higher *oβ–2R* expression levels (Kaku et al., 2025). Previous laboratory-based studies demonstrated that precocious foraging may be induced by OA supplementation (Schulz and Robinson, 1999).

Honey bees are exposed to environmental OA sources including citrus blossom nectar, which may contain tyramine and OA at concentrations up to 256 µM (Muth et al., 2022; Barberis et al., 2023b; Barberis et al., 2023a; Tang et al., 2006). In addition, acaricides that are OA receptor agonists including amitraz and chlordimeform are commonly used in agriculture (Ahmed and Vogel, 2020). Amitraz is commonly used to mitigate *Varroa* mite infestations in honey bee colonies (Guo et al., 2021). *Varroa* mites with amitraz-resistant Oβ–2R mutations have been reported (Rinkevich et al., 2023; Hernández-Rodríguez et al., 2025). Amitraz has generally been considered safe for honey bees since honey bee OA receptors are divergent from *Varroa* mite OA receptors (i.e. 48% identical at the amino acid level; Guo et al., 2021; National Center for Biotechnology Information (NCBI), 2024). However, honey bees exposed to amitraz had higher mortality when co-stressed via infection with a model virus (Flock House virus; O’Neal et al., 2017). These results, in conjunction with our findings linking virus infection with the OA stress response pathway, may indicate previously unrecognized impacts at the organismal level. *Varroa* mites vector many honey bee viruses, and infested colonies often have coinfections and greater average virus levels (Doublet et al., 2024). Therefore, the application of miticide to reduce *Varroa* and control vectored viruses, or the accumulation of miticide metabolites in wax or other hive matrices, may inadvertently harm co-stressed, virus-infected honey bees (O’Neal et al., 2017; Bischoff et al., 2023; Mullin et al., 2010). Collectively, the data presented herein demonstrate that the OA pathway is a key factor in mediating virus-specific effects on honey bee flight performance, which may in turn influence viral transmission and colony health.

## Materials and methods

**Key resources table:** 

Reagent type (species) or resource	Designation	Source or reference	Identifiers	Additional information
Chemical compound, drug	epinastine (EP)	Tokyo Chemical Industry	C_16_H_15_N_3_ x HCl	EP-hydrochloride >98%
Chemical compound, drug	octopamine (OA)	Thermo Fisher Scientific	C_8_H_11_NO_2_ x HCl	OA-hydrochloride 99%
Gene (*A. mellifera*)	*rpl8*	NCBI GeneID: 410188	GB48810	(*A. mellifera*) *ribosomal protein L8*
Gene (*A. mellifera*)	*oβ–2R*	NCBI GeneID: 412896	Octbeta2R, GB49696, Octbeta2	Gene (*A. mellifera*) *OA β–2 receptor*
Gene (*A. mellifera*)	*tdc*	NCBI GeneID: 410947	Tdc1, GB55831	Gene (*A. mellifera*) *tyrosine decarboxylase*
Gene (*A. mellifera*)	*tβh*	NCBI GeneID: 751102	AmTbH, GB16337, GB53665	Gene (*A. mellifera*) *tyramine β-hydroxylase*
Other	Honey bee	NCBI SRA: PRJNA1414634	Illumina sequencing of RNA obtained from virus-infected honey bees no- or post-flight, Jan 23 '26	*A. mellifera* transcriptome data

### Honey bees

Honey bees (*Apis mellifera,* primarily *carnica*)*,* were maintained with standard apicultural practices at Montana State University’s Horticulture Farm. Each experiment was performed with honey bees from different colonies to obtain robust results from genetically diverse bees (Source data 1, data S1). Frames with newly emerging bees were collected one day prior to each experiment and maintained at 32 °C in a laboratory incubator overnight. Young, age-matched (~24 hr post-emergence), female adult bees were utilized for all experiments. Bees were cohoused in modified deli containers (n=10 per house), maintained at 32 °C in an incubator, and fed 50% sucrose syrup and water ad libitum (Brutscher et al., 2017). All honey bees were fed only sucrose syrup unless OA and/or epinastine (EP) oral treatment was otherwise specified. OA was administered at a concentration of 10 mM and EP at 4 mM in fresh sucrose syrup daily, and honey bees were given unrestricted access to the supplemented sucrose syrup for the duration of the experiment.

### Virus stock preparation

Deformed wing virus (DWV) and sacbrood virus (SBV) inoculums were propagated in white-eyed pupae as previously described (Kaku et al., 2025). In brief, pupae were injected with 2 µL of Tris-HCl (pH 7.5) containing either 4.0 × 10^6^ DWV RNA copies or 3.5 × 10^4^ SBV RNA, between the second and third abdominal tergites using a Harbo syringe and a disposable borosilicate needle made from modified capillary tubes. Pupae were maintained in individual wells in a 24-well plate with a piece of Whatman filter paper at the bottom of each well to prevent pupal tissue damage. In each plate, four wells were filled with sterile water for humidity, at 32 °C for 7 days. Pupae were then homogenized in a 2 mL microfuge tube with a sterile, metal, 3 mm bead in 1 mL 1 X PBS (pH 7.4) using the TissueLyser II (Qiagen) at 30 Hz for 2 min. Homogenates were centrifuged at 14,000 × *g* for 15 min at 4 °C and supernatant was transferred to a fresh tube. The virus-containing lysates were filtered through a 0.45 µm filter, then a 0.22 µm filter to remove large particles and non-viral microbial contaminants, respectively. RNA was isolated from the virus-containing filtrate (20 µL) using TRIzol (Thermo Fisher) according to the manufacturer’s instructions. RNA was quantified using a NanoDrop 2000c Spectrophotometer, cDNA was synthesized with reverse transcriptase, and virus RNA copies (including both genomes and transcripts) were quantified from cDNA using qPCR. The inoculum was tested for copurifying/contaminating viruses via PCR (Source data 1, data S2, Appendix 1—figure 2). RNA copy concentration in the purified virus stock was quantified relative to a standard curve using qPCR (Source data 1, data S3). DWV genome consensus sequences were assembled from short-read sequencing data obtained from sequencing RNA isolated from the inoculum (DWV-lab 2024; GenBank PV821422) and SBV sequencing data (SBV isolate MT 2023; GenBank PV788228.1). Virus inoculum aliquots were stored at –80 °C until each experiment.

### Experimentally introduced honey bee virus infections

Honey bees were infected with DWV using previously described methods (Brutscher et al., 2017). In brief, age-matched bees were cold anesthetized at 4 °C for 10 min and infected via intra-thoracic injection using a Harbo syringe with 3.5 × 10^4^ DWV RNA copies, 3.5 × 10^4^ SBV RNA copies, or coinfected with 3.5 × 10^4^ DWV RNA copies and 9.1 × 10^3^ SBV RNA copies. If OA and/or EP treatment was administered via injection, the injection inoculum also contained 10 mM OA and/or 4 mM EP so that each bee only experienced one injection to reduce injection-associated stress. Each inoculum dose was suspended in 2 µL10 mM Tris-HCl buffer, pH 7.5. Buffer injected/mock-infected bees were injected with 2 μL buffer. The DWV doses utilized in this study were similar to previous studies and resulted in virus levels commonly found in naturally infected bees (Traynor et al., 2016; Wells et al., 2016; Faurot-Daniels et al., 2020; D’Alvise et al., 2019; Glenny et al., 2017; Runckel et al., 2011). As previously described (Kaku et al., 2025), bees had #000 steel washers affixed to their thoraces using rubber cement immediately after injections; washers had an outside diameter of 0.19 cm and weighed 4.3 mg, less than half of an average pollen load (Winston, 1991).

### Flight mill

The flight mills used in this study were based on flight mills used in previous honey bee studies (Kaku et al., 2025; Brodschneider et al., 2022; Tosi et al., 2017; Scheiner et al., 2013) and included a magnet to tether bees from the affixed steel washer on each thorax, which enabled their removal from flight mills with minimal damage. Flight assays were performed as previously described (Kaku et al., 2025). In brief, bees 72 hpi (i.e. 4 days post eclosion) were immobilized via incubation at 4 °C for ~12 min and tethered to flight mills; counterweights were adjusted for each bee. Temperature and humidity were maintained the same for each experiment (23 °C and 31 ± 4% relative humidity). Flight was instigated by tapping the flight arm downward and was re-initiated up to three times before bees were considered ‘exhausted’ and the data collected was representative of ‘total flight’ capability (Source data 1, data S1- Data S1). Bees were stored at –80 °C immediately after flight. Flight distance, duration, and speed calculations were based on redlight detector counts which were recorded on an attached Raspberry Pi as previously described (Kaku et al., 2025). Active flight duration was calculated as the sum time moving >5.5 cm (one encoder count; Source data 1). Flight stop count was defined as any stop that exceeded 1.5 s. Honey bees may stop to seemingly engage in grooming behaviors; stops were counted if the bee re-initiated flight (Kaku et al., 2025). DWV-infected, EP-injected, SBV-infected bees fed OA or EP or injected with OA, or SBV-infected bees fed both OA and EP had fewer stops than mock-infected bees (Source data 1, data S4).

### Honey bee RNA isolation

RNA was isolated from the abdomen of each individual honey bee since abdomens were representative of the entire bee (Kaku et al., 2025). Each abdomen was individually homogenized in sterile water (300 μL) with one sterile steel bead (4.5 mm) using a Qiagen TissueLyser for 2 min at 30 Hz. Lysates were centrifuged at 4 °C for 5 min at 7500× *g*. RNA was isolated from the supernatants using equal volumes of TRIzol reagent according to manufacturer’s instructions. The concentration and quality of RNA samples were assessed on a Thermo Fisher Nanodrop 2000 spectrophotometer. RNA was stored at −80 °C.

### Reverse transcription/cDNA synthesis

Reverse transcription reactions were performed by incubating 2 μg RNA, 200 U M-MLV reverse-transcriptase, and 500 ng random hexamer primers in 25 μL reactions for 1 hr at 37 °C, according to the manufacturer’s instructions. cDNA was diluted with sterile water (1:2) and 2 μL were used for PCR.

### Polymerase chain reaction

Since bees were obtained from colonies that are subject to natural infections (Faurot-Daniels et al., 2020; Brutscher et al., 2017), PCR was performed to test for preexisting virus infections. Specifically, 2 μL cDNA template was combined with 10 pmol of each forward and reverse primers (Source data 1, data S2) and amplified with ChoiceTaq polymerase according to the manufacturer’s instructions using the following conditions: 95 °C for 5 min; 95 °C for 30 s, 57 °C for 30 s, 72 °C for 30 s, 35 cycles, followed by final elongation at 72 °C for 4 min. PCR products were assessed by gel electrophoresis (1.5% agarose with SYBR safe) and visualized using a Syngene U:Genius 3 imaging system (Appendix 1—figure 1). PCR products were previously verified by sequencing (Faurot-Daniels et al., 2020). Analysis of pooled cDNA samples from mock-infected honey bees from each of these experiments revealed that many bees had preexisting DWV and SBV infections; therefore, qPCR was used to assess their abundance in all individual samples (Source data 1, data S1). The qPCR primers used in this study to target DWV, bind to both DWV-A (GenBank AY292384) and DWV-B (GenBank MN565036), in addition to numerous other DWV sequences on NCBI including the consensus sequences of the DWV inoculum as part of this study (Datal S3).

### Quantitative PCR

Quantitative PCR (qPCR) was used to assess virus abundance and relative abundance of honey bee transcripts. All qPCR reactions were performed in triplicate with 2 μL of 1:2 diluted cDNA template. Each reaction contained 1×ChoiceTaq Mastermix, 0.4 μM each forward and reverse primer, 1×SYBR Green (Life Technologies), and 3 mM MgCl_2_ for a total volume of 20 μL per reaction. A CFX Connect Real Time instrument (Bio-Rad) was used for the following: pre-incubation 95 °C for 1 minute followed by 40 cycles of 95 °C for 10 s, 58 °C for 20 s, and 72 °C for 15 s, with a final melt curve analysis at 65 °C for 5 s to 95 °C. To quantify virus copies in each sample, plasmid standards (i.e. virus-specific PCR amplicons cloned into the pGEM-T (Promega) vector and sequence verified) for each virus were used as templates, ranging from 10^3^ to 10^9^ copies per reaction for each standard curve as previously described (Source data 1, data S3; Kaku et al., 2025). The honey bee gene *rpl8* was used as a housekeeping gene for each sample for comparison and for relative fold change calculations (Source data 1, data S1 and S2). Sterile water containing no DNA template was used for negative controls. qPCR specificity was verified by melt point analysis, gel electrophoresis, and sequencing (Faurot-Daniels et al., 2020). The starting quantity (SQ) of cDNA template was calculated for each honey bee sample as previously described (Kaku et al., 2025). For each honey bee sample, the starting quantity (SQ) of cDNA template (representing 80 ng total RNA) was calculated based on the standard curve and subtracting the average SQ of the no template control reaction (<600 copies). Samples with virus RNA copies below the limit of detection for qPCR (<1000 copies/2 µg RNA) were listed as 0 (Source data 1, data S1). Virus abundance was reported as virus RNA copies, including genomes and transcripts, per 2 µg total RNA (i.e. per RT reaction) and ranged up to 7 × 10^10^ virus RNA copies/2 µg RNA. Whole bee data was estimated based on the previous estimate of 138 µg RNA per whole bee (Kaku et al., 2025). Comparison of virus abundance levels using estimated copy numbers enabled inclusion of values of 0. Using the ΔΔCt method to calculate virus levels in bees with no detectable virus would inaccurately result in ‘missing’ data and consequently skew model interpretations. Relative host gene expression was calculated using the average ΔΔCt method where ΔCt was determined by subtracting *rpl8* Ct from the gene of interest Ct. Average ΔCt was calculated by the mean ΔCt of the buffer-injected bees in each experiment, which harbored the lowest virus levels. The ΔΔCt was calculated by subtracting the average control ΔCt and fold change was determined by the equation 2^-ΔΔCt^ (Source data 1, data S3).

### RNA sequencing and analysis

Honey bee samples with low/no preexisting infections (n=3–5) were selected per treatment from previously published data (Kaku et al., 2025; n=36 total) (Source data 1, data S5). RNA from each sample was DNase treated using Qiagen RNeasy columns. RNA samples were sent to the Roy J. Carver Biotechnology Center at University of Illinois; quality was assessed with an Agilent 3500 fragment analyzer. Libraries were prepared via Watchmaker polyA RNA-Seq prep kit according to manufacturer instructions. Libraries were sequenced on an Illumina NovaSeq X Plus 25B (paired 150 nt reads), approximately 3.2 billion total paired end reads were obtained. Sequencing quality of reads representing individual samples using FastQC, and filtering of the comprehensive dataset was done using MultiQC versions 0.12.1 (Andrews, 2010). Illumina adaptors were trimmed and low quality reads were omitted when Q<30 with BBduk from BBTools version 39.26 (Bushnell, 2014). Reads were normalized with shrinkage estimations for dispersion using DESeq2 l (correction with q-value<0.05) (Love et al., 2014). Sequencing libraries were aligned to the *A. mellifera* genome, Amel_HAv3.1 (i.e, GCA_003254395.2_Amel_HAv3.1_genomic.fna, downloaded from NCBI 2019–02–07) (Weinstock et al., 2006; Elsik et al., 2014). Sequencing data from individual samples were uploaded to the NCBI short read archive (SRA) (BioProject ID: PRJNA1414634). Genes were considered differentially expressed if log_2_ fold change of transcript abundance had adjusted p-values <0.05. Venn diagrams were produced using the eulerr package in R (Larsson and Gustafsson, 2018).

For virus quantification, trimmed paired end read files were aligned to a nonredundant version of the Holobee database (i.e. https://doi.org/10.15482/USDA.ADC/1255217; Evans et al., 2016) which considered 32 potential virus alignments, using HISAT2 version 2.2.1 (Kim et al., 2019). Default parameters were used during alignment to allow multimapping of reads. Samples with unexpected infections were omitted from analyses (n=6/36; Source data 1, data S5). Alignment files were converted to compressed alignment files and sorted using samtools version 1.21 and unmapped reads were discarded (Li et al., 2009). The samtools built-in idxstats command was used to quantify virus abundance in sorted alignment files and generate read counts. Read counts of each trimmed FastQ file were quantified using the Seqkit stats command (Shen et al., 2016). Computational efforts were performed on the Tempest High Performance Computing System, operated and supported by University Information Technology Research Cyberinfrastructure (RRID:SCR_026229) at Montana State University.

### Statistics

Statistical analyses were performed in R 4.3.3. Log-transformations and non-parametric tests were performed as necessary. Specifically, all relative gene expression data was log_2_ transformed and all virus abundance data were log_10_(1+) transformed, which enabled inclusion of zero. All mixed models were assessed using the lmer4 package (Bates et al., 2015). Assumptions for linear models were verified by diagnostic plots and histograms using base R functions and the performance package (Lüdecke et al., 2021; Schielzeth et al., 2020). All models included experiment ID as a random effect and where relevant, virus injections and flight mill IDs were also included as random effects (Source data 1, data S4). Models were compared using corrected Aikike information criterion (AICc) and Bayesian information criterion (BIC). Models were assessed via maximum likelihood (ML) when random effects varied and via restricted maximum likelihood (REML) when fixed effects varied. We reported only results from the best model for each analysis, but other considered models were included in supplementary material (Source data 1, data S4). For each linear mixed model with reported estimates, p-values were estimated from *t*-statistics via Satterthwaite’s method (Kuznetsova et al., 2015).

### Figures

All figures were generated through GraphPad Prism software for Windows using version 10.6.0, in RStudio using version R 4.3.3, in BioRender, and Adobe Illustrator.

## Figures and Tables

**Appendix 1—figure 1.**
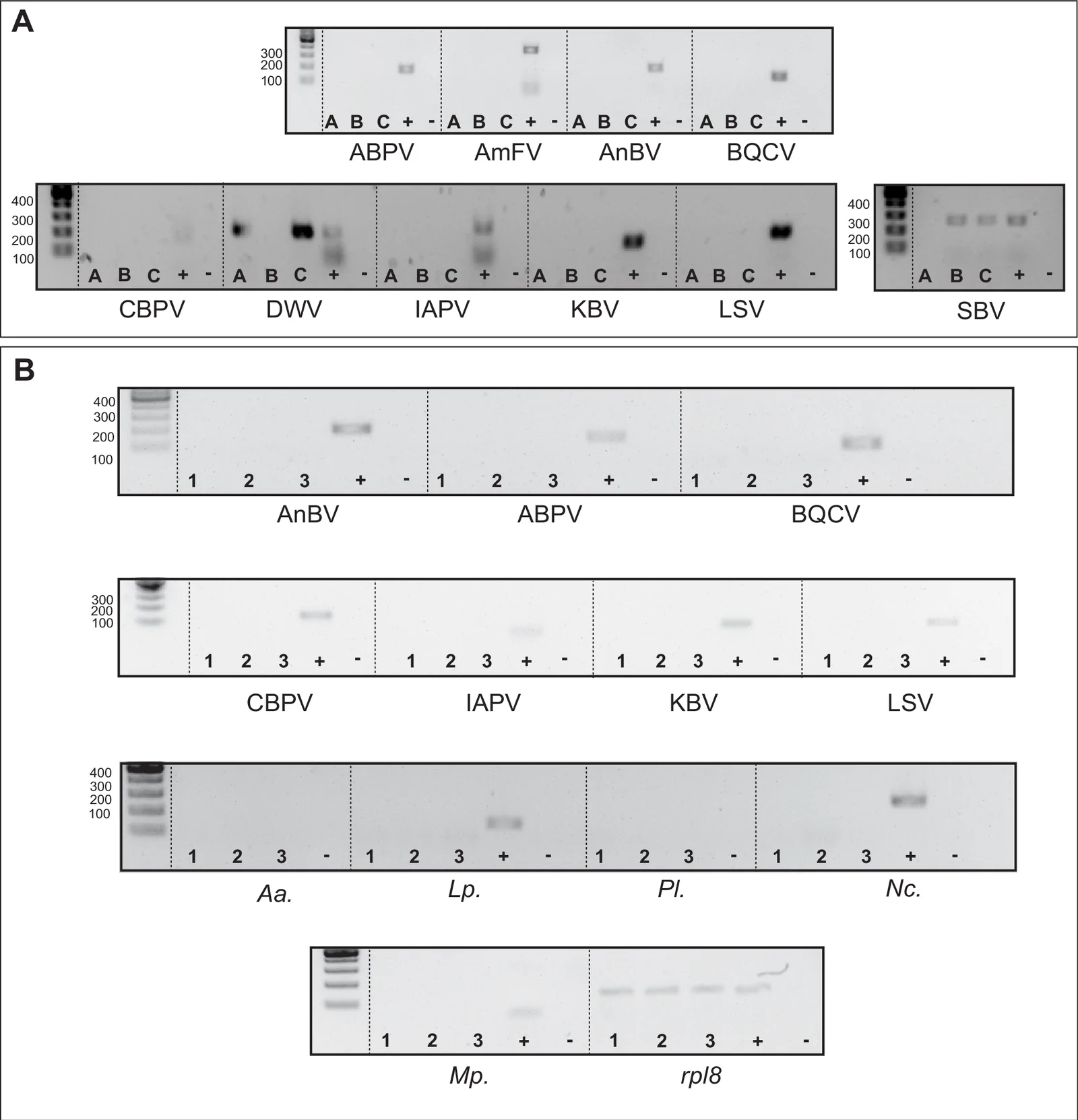
Pathogen testing of virus inoculum and honey bee samples. (**A**) Filtered virus inoculums DWV, SBV, and DWV + SBV (labeled A, B, or C, respectively) were tested for other common viruses including acute bee paralysis virus (ABPV), *Apis mellifera* filamentous virus (AmFV), Andrena bee-associated virus-1 (AnBV), black queen cell virus (BQCV), chronic bee paralysis virus (CBPV), deformed wing virus (DWV), Israeli acute paralysis virus (IAPV), Kashmir bee virus (KBV), Lake Sinai viruses 1–4 (LSV), and sacbrood virus (SBV) using virus-specific polymerase chain reaction (PCR) and analyzed by gel electrophoresis; positive (+) and negative (-) controls. (**B**) Pathogen diagnostic PCR was performed using pooled mock-infected honey bee cDNA from experiments 1–3 (labeled 1–3) as template determined that bees had preexisting DWV (experiments 1–3) and SBV (experiments 1–3). All samples were tested for DWV and SBV via qPCR (Source data 1, data S1Data S1). All experiments were negative for all non-viral pathogens including *Ascosphaera apis* (*Aa*.), *Lotmaria passim* (*Lp*.), *Melisococcus plutonius* (*Mp*.), *Nosema ceranae* (*Nc*.), and *Paenibacillus larvae* (*Pl*.); cDNA quality was assessed via amplification of the honey bee housekeeping gene, rpl8. Although no positive control was available for *A. apis* or *P. larvae*, the primers were utilized successfully in previous studies. To illustrate this result in a single figure, cDNA from mock-infected individual bee samples from all experiments were pooled by experiment (i.e. n=12 from experiment 1, n=12 from experiment 2, n=12 from experiment 3) and PCR was repeated. The products of pathogen-specific PCRs using pooled cDNA samples, positive (+), and negative control (-) templates were analyzed by agarose gel electrophoresis. Appendix 1—figure 1—source data 1.Original gels. Appendix 1—figure 1—source data 2.Labelled gels.

**Appendix 1—figure 2.**
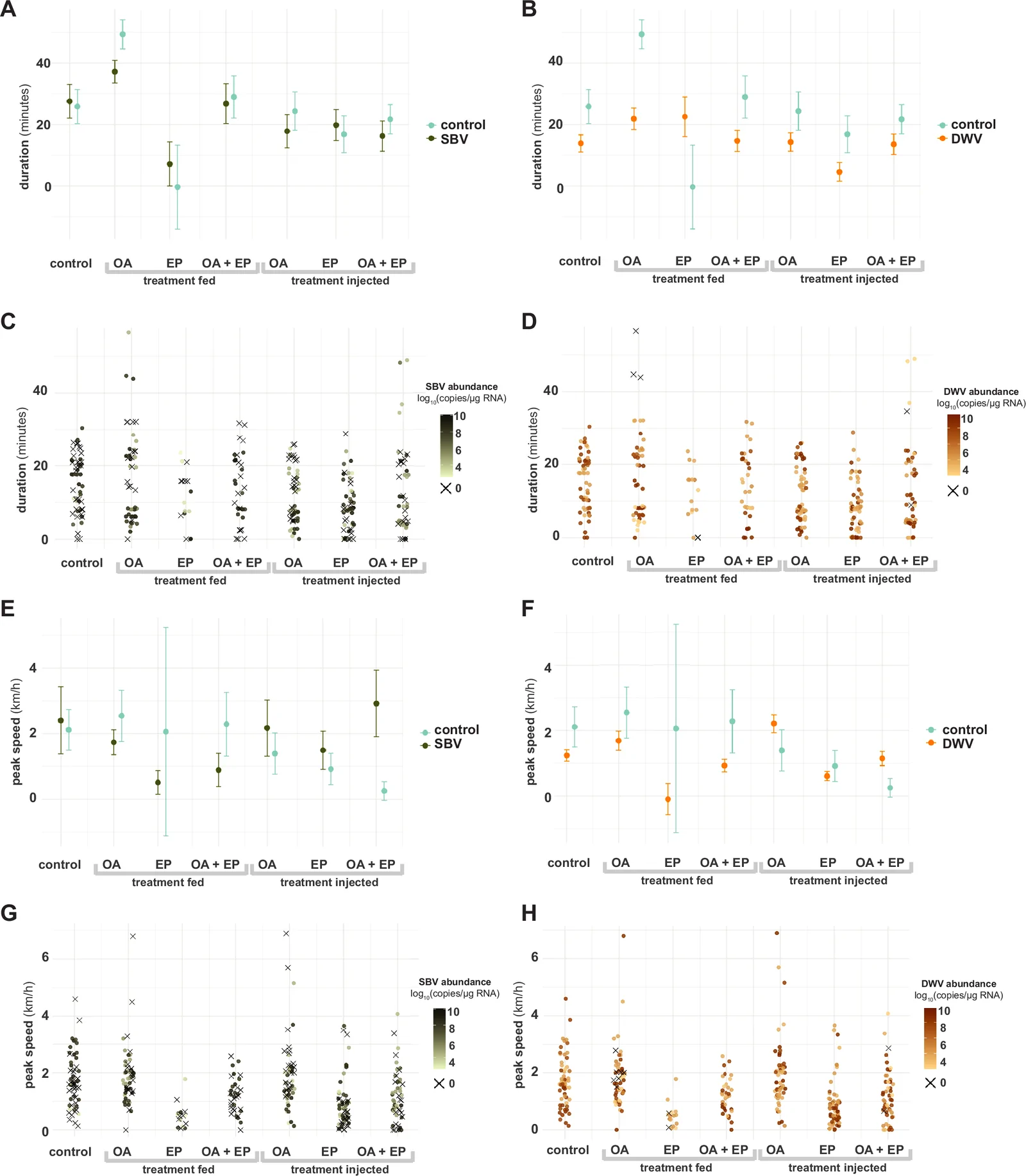
Estimated flight duration and speed by virus and OA and/or EP treatment To visualize the effects of virus infection and octopamine (OA) and/or epinastine (EP) treatment on honey bee (**A**–**D**) flight duration (minutes) and (**E**–**H**) peak flight speed (km/h), we compared estimated means using experimental data analyzed with linear mixed models (Source data 1, data S4). Each individual point represents the estimated marginalized means with bars indicating one standard deviation of the mean. Green points represent predictions when bees harbored high SBV levels (i.e. 108 SBV RNA copies/2 μg RNA) and orange points represent predictions when bees harbored high DWV levels (i.e. 108 DWV RNA copies/2 μg RNA). Blue points represent estimates for virus-free bees. (**A**) SBV-infected bees flew similar durations to uninfected bees, and all treatments resulted in similar or shorter flight durations than those fed sucrose only except OA-fed bees, which flew for greater durations. (**B**) DWV-infected bees flew for shorter durations than uninfected bees, but EP and OA treatment resulted in greater flight durations. (**C**, **D**) Observed flight durations where each point represents data collected from an individual bee (total n=336) and the color scale represents virus levels. (**E**) SBV-infected bees flew similar peak speeds to uninfected bees, but any OA or EP treatment resulted in lower peak speeds except for OA +EP injected and OA injected bees, which flew similar distances to those fed only sucrose. (**F**) DWV-infected bees flew at slower speeds than uninfected bees. When DWV-infected bees were fed or injected with OA, peak speeds were slightly higher. (**G, H**) Observed peak flight speeds where each point represents data collected from an individual bee with (**G**) SBV infections or (**H**) DWV infections.

**Appendix 1—figure 3.**
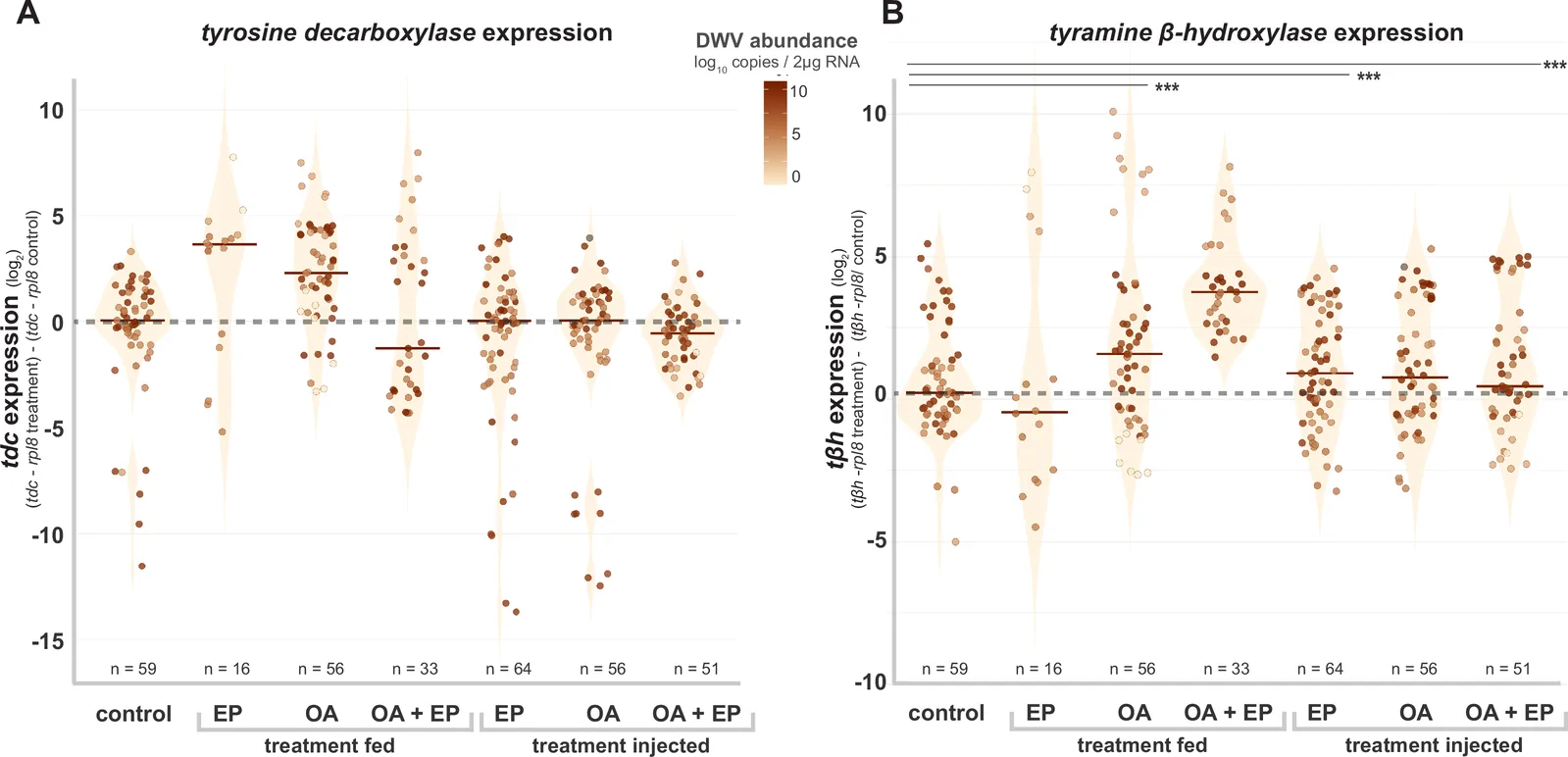
DWV-infected bees had no relationship with *tdc* and treatment-specific relationships with *tβh.* To identify relationships between *tyrosine decarboxylase* (*tdc*) and *tyramine β-hydroxylase* (*tβh*) expression across virus infection and treatments, we evaluated relationships using a linear mixed effect model (Source data 1, data S4). To visualize the data, we plotted all data points against treatment groups with either (**A**) *tdc* expression or (**B**) *tβh* expression. The background violin plots represent the 95% confidence interval, the individual data points represent individual bee data, the horizontal line in each violin represents the median for the treatment group. Ranges of DWV infection were included as a color scale (i.e. darker orange representing higher DWV infection levels). If there is no effect of DWV infection, the darkest points would be evenly distributed above and below the control ‘0’ fold change line. SBV levels were more strongly associated with *tdc* expression (Figure 3). There was no difference in *tdc* expression by DWV abundance (p=0.83). (**B**) The expression of *tβh* was greater in DWV-infected bees relative to uninfected bees (p<0.0001, Source data 1, data S4). In addition, DWV-infected bees fed OA, injected with EP, or injected with OA and EP exhibited reduced expression (p-values <0.001, Source data 1, data S4).

**Appendix 1—figure 4.**
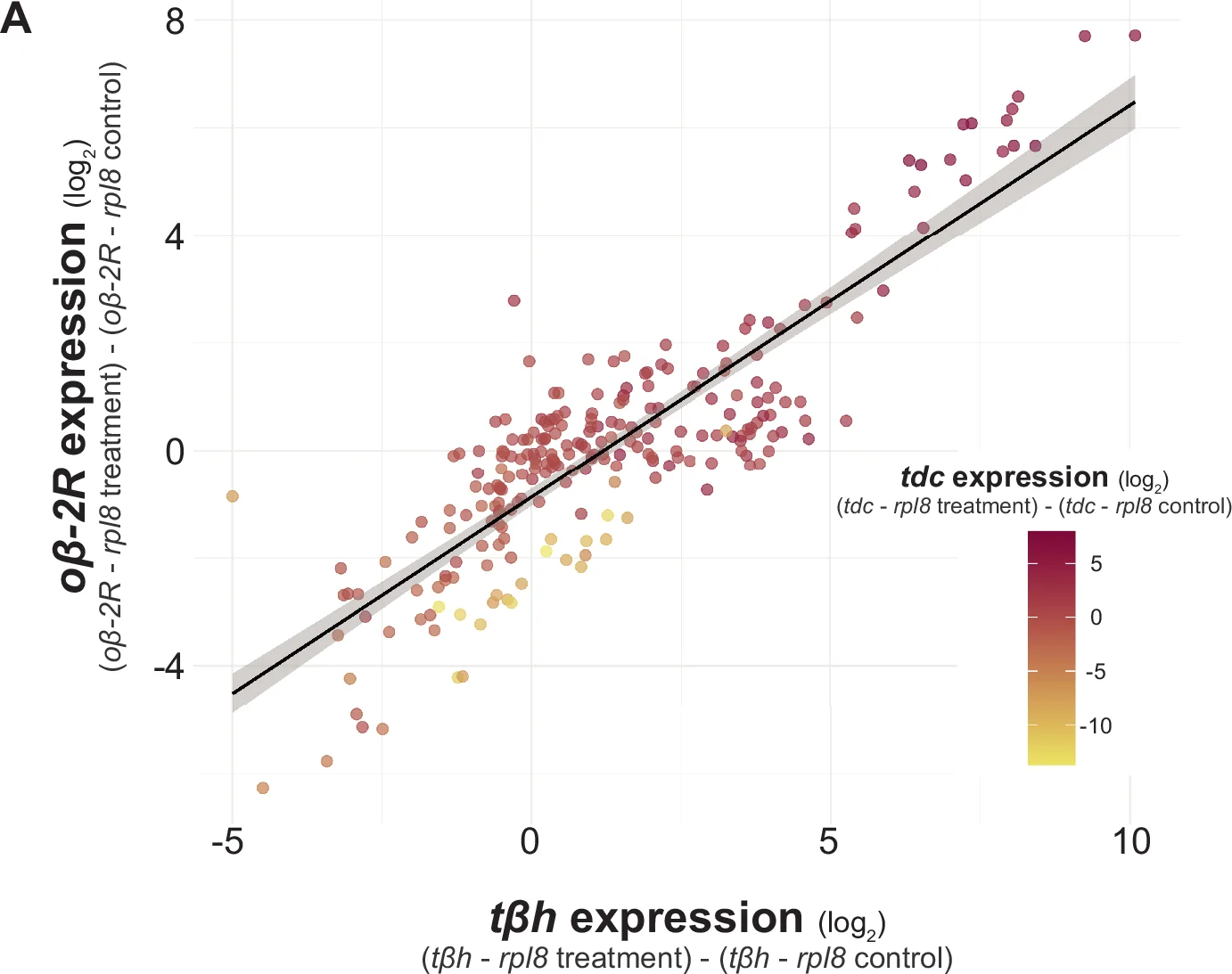
Expression of tdc, tβh, and oβ–2R are positively correlated The relationships between the expression of tdc, tβh, and oβ–2R in honey bees were evaluated using a linear mixed effect model that included tdc, tβh, and oβ–2R expression, and SBV abundance as fixed effects and experiment, injection, and treatment (i.e. OA and/or EP) as random effects. This model, which explained 95% of the data, determined that *tdc*, *tβh*, and SBV abundance were associated with increased *oβ–2R* expression (p-values <0.05, SI Source data 1, data S4). Each data point represents data from an individual honey bee across three experiments (total n=336; Source data 1, data S1 and S9Data S1 and S9).

**Appendix 1—figure 5.**
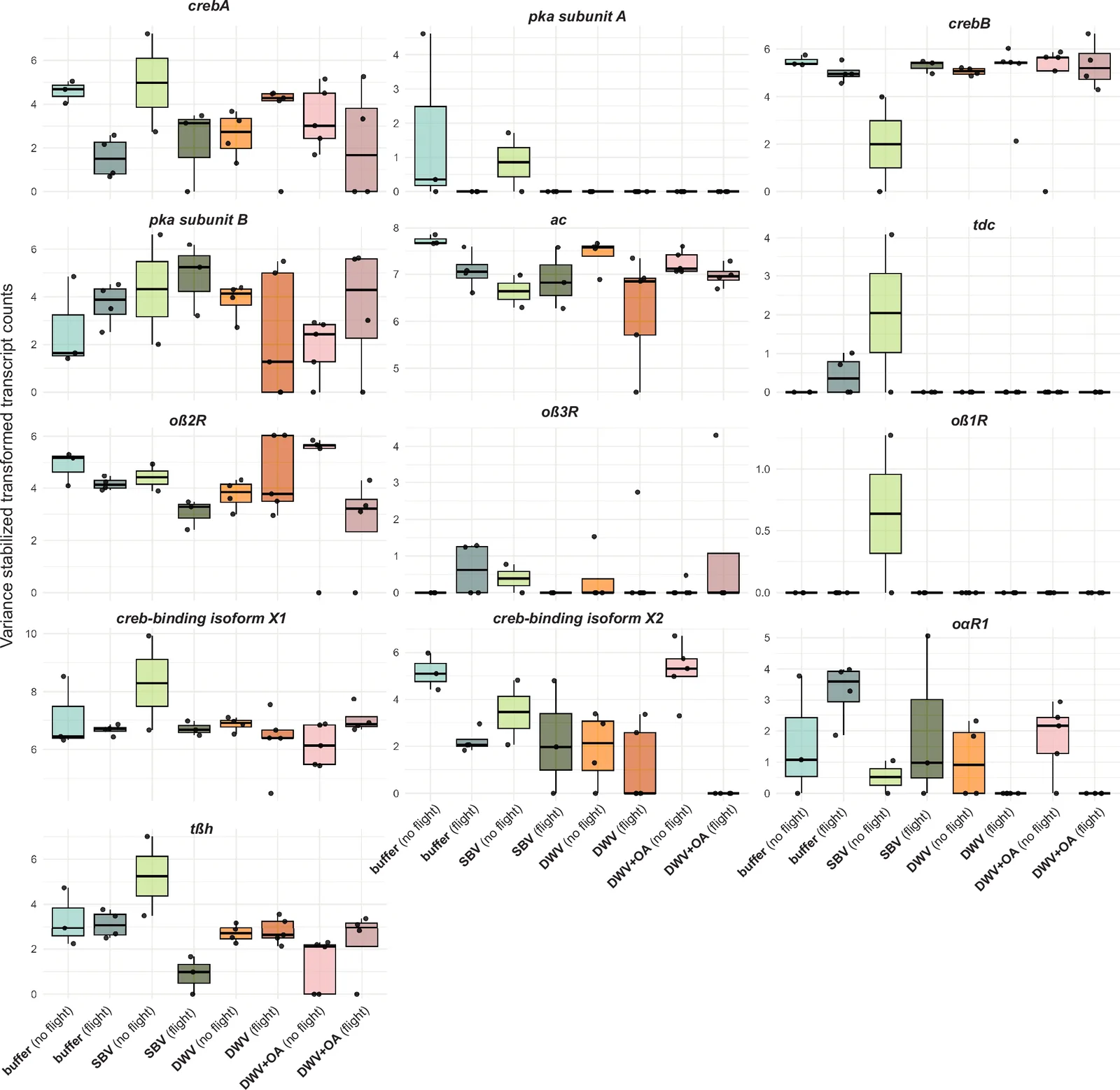
Comparison of honey bee OA-associated gene expression To identify potential differences in expression between different treatments and octopamine (OA)-associated genes, expression (as variance stabilized transformed values) between different treatments were evaluated. Specifically, the expression of creb subunits (**A **and **B**), *protein Kinase A* (PKA) subunits, OA receptors (*oβ–2R*, *oβ–3R*, *oβ–1R*, and *oα–1R*), *adenylyl cyclase* (*ac*) and the two precursor enzymes that convert tyrosine to tyramine and tyramine to OA (i.e. *tdc* and *tβh*, respectively). Each point represents data from an individual bee. Blue boxplots were mock infected with buffer injections, green boxplots were experimentally infected with sacbrood virus (SBV), orange boxplots were experimentally infected with deformed wing virus (DWV), and pink boxplots were coinjected with DWV and OA (DWV +OA). Lighter colors indicate treatments that did not fly and darker color boxplots indicate treatments that flew (see Figure 5). We compared the average expression of OA associated genes across treatment groups and determined that SBV-infected bees that did not fly had the greatest differences in OA-associated gene expression relative to all other treatment groups (Complete data available in Source data 1, data S9).

## Data Availability

All data are available in the main text and source data files. Sequencing data from individual samples were uploaded to the NCBI short read archive (SRA) (BioProject ID: PRJNA1414634). The following dataset was generated: KakuNG FlennikenML 2026Next generation sequencing of virus-infected honey bees no- or post-flightNCBI BioProjectPRJNA1414634

## Additional files

MDAR checklist

Source data 1. Individual honey bee data. Data S1. Individual honey bee data. To compare the relationship of the octopamine response in virus-infected bees, we performed three experiments. Bees were injected with buffer, DWV (3.5 × 10^4^ copies), or SBV (3.5 × 10^4^ copies). Some bees were coinjected with octopamine (OA), epinastine (EP), OA and EP, or only received the virus/buffer injections. All bees were fed sucrose syrup (50% weight/volume); some sucrose syrups were supplemented with OA, EP, or OA and EP. Bees were cohoused (8-10 bees/house) and maintained in laboratory conditions for 72 hpi prior to flight. At 72 hpi, bees were magnetically tethered to flight mills where flight performance (i.e. flight speed, flight distance, flight stop count) were recorded. For a subset of bees, post-flight temperatures were recorded (F). Immediately post-flight, bees were stored at −80°C before samples were processed. Each bee was weighed post-flight, virus was quantified via qPCR (see data sheet S3). All samples were tested for DWV and SBV via qPCR (columns M-R), and 12 samples per experiment were pooled and tested for an additional 13 potential preexisting pathogens via end-point PCR (columns S-AE). Data S2. Primers used in this study. Data S3. Standard curves for qPCR virus quantification (DWV, SBV, and IAPV). Quantification of virus abundance in samples was assessed using the standard curves generated from using known quantities of plasmid standards as template DNA on each qPCR plate with samples; representative standard curves are shown below. The copy number per well or ‘starting quantity’ (SQ) value calculated in no template control reactions (<1,000 copies) was subtracted from each standard SQ value. For standardization, the qPCR instrument baseline threshold was set to 1000 for all plates. Data S4. Best fit linear mixed models with estimates, standard error (SE), p-values, and conditional R2 values. Linear mixed models were used to assess honey bee flight distance (log_10_ m), average flight speed (km/hr), peak flight speed (km/hr), flight stop count, active flight duration (seconds), *tyrosine decarboxylase* (*tdc*) expression, *tyramine beta-hydroxylase* (*tbh*) expression, *octopamine receptor* (*oβ-2R*) expression, and comparisons of all mixed models as assessed via REML or ML comparing AICc and BIC values. Data S5. HoloBee virus alignments. Sequenced honey bee samples were aligned to the HoloBee v2016.1 genome alignment; virus alignments included in this sheet including DWV and SBV. Additionally, qPCR estimates included in columns AM-AN for DWV and SBV infections. This sheet also includes NCBI accessions for virus genomes considered in these alignments. SBV was aligned to the SBV stock experimentally introduced in these infections (in column AJ). Values with <1000 reads were not considered active infections for the purpose of sample analysis. Only four samples were omitted (highlighted in orange) due to unexpected preexisting infections. Data S6. Differentially expressed genes in bees with either DWV, SBV, or DWV and OA (DWV+OA) injections post-flight, including the log2 fold change relative to mock-infected bees post-flight. Adjusted for multiple comparisons using Benjamini-Hochberg correction on p-values. Data S7. Immune gene expression. To compare expression of a subset of immune genes, variance stabilized transcript count was determined in each individual sample, and log_2_ fold change of conditions were analyzed relative to mock-infected bees in either post-flight or no-flight conditions. Sample numbers correspond to sample IDs in Appendix 1, data S5. When fold change was significantly different, Benjamini-Hochberg p-values were highlighted in green. Post-flight sample columns are blue and no-flight sample columns are gray. Data S8. Expression of all genes post-flight relative to mock-infected honey bees. Bees were experimentally infected with SBV, DWV, or coinjected with DWV and octopamine (OA). To correct for multiple comparisons, p-values were adjusted with Benjamini-Hochberg corrections. Data S9. Comparison of a subset of octopamine (OA) pathway associated genes. To assess differences in genes associated with the OA response across different treatments, we identified a sublist of 13 genes. Since we wanted to compare expression across each group and not relative to any baseline group (such as with differential gene expression analyses), we assessed variance stabilized transformed sequence counts. These data are plotted to identify trends in Appendix 1—figure 1. Data S10. 30 genes with greatest normalized mean expression difference in DWV+OA injected bees relative to DWV only injected bees. To identify genes important to DWV+OA injected bees, we identified 50 genes with the greatest difference in DWV+OA injected bees relative to DWV-only injected bees for bees post-flight and bees that were not flown; the two lists of 50 top genes shared 30 genes, presented here. Using NCBI DAVID functional annotation, genes from this list were enriched for signaling (p = 0.0000007), antimicrobial function (p = 0.0009), mitochondrial function (p = 0.005), and chitin binding (adjusted p-value = 0.003). Expression values were not used for evaluating statistically significant differences in expression, but for identifying a subset of genes for further analyses.
