# Supplementary material for: Virus-specific impacts on honey bee flight performance are mediated by the octopamine pathway
Source: eLife. 2026 Nov 9;15:RP111072. doi: 10.7554/eLife.111072 (PMC13747321; doi:10.7554/eLife.111072)
Supplement: Peer review history

# Editor report: eLife Assessment

**DOI:** 10.7554/eLife.111072.3.sa0

**Authors:** John W Schoggins

**Affiliations:** The University of Texas Southwestern Medical Center United States

**Keywords:** Important, Solid

This **important** study investigates how distinct honeybee viruses differentially alter flight performance through interactions with octopamine signaling pathways. The combination of behavioral flight assays, pharmacological perturbation, and transcriptomic analyses provides **solid** evidence that virus-specific effects on flight are associated with octopamine signaling. The data presented also establish a framework for additional analyses that may strengthen the proposed mechanistic model, including quantification of endogenous octopamine levels and receptor specificity studies.

# Referee report: Reviewer #1 (Public review)

**DOI:** 10.7554/eLife.111072.3.sa1

Summary:

Kaku and Flenniken investigate the mechanistic pathways through which specific viral infections alter the flight capabilities of honeybees. Building on their previous discovery that DWV impairs flight while SBV unexpectedly enhances it, the authors hypothesized that these behavioral shifts are driven by interactions with the insect's octopamine (OA) signaling pathway, which is responsible for the "fight-or-flight" neurohormonal stress response and energy mobilization. To test this, the authors experimentally infected adult honeybees with DWV or SBV and pharmacologically manipulated the OA pathway using either octopamine supplementation or epinastine (EP), an OA-receptor antagonist. They then evaluated the bees' flight performance (distance, duration, and speed) on custom flight mills and profiled their gene expression using qPCR and RNA sequencing.

Strengths:

A major strength of this study Is the high prevalence of preexisting background DWV and SBV infections in the honeybee cohorts, which meant there were no completely "virus-free" control groups. However, the authors successfully mitigated this limitation by rigorously quantifying viral RNA copies for every individual bee via qPCR and utilizing these viral abundances as continuous variables in powerful linear mixed-effect models.

Weaknesses:

The primary weakness lies in the methodology used for targeted pharmacological manipulations, as well as the lack of OA quantification across different treatments. Thus, their claims are not sufficiently supported by the current data.

Comments on revised version.

I appreciate the authors' efforts to address the reviewers' concerns and to revise the wording of the manuscript. The revised version is more cautious than the original, and some of the discussion has been appropriately toned down. However, I remain unconvinced that the key mechanistic conclusions are sufficiently supported by the current evidence.

(1) The specificity of epinastine remains insufficiently demonstrated.

The authors argue that AmOARβ2 is the predominantly expressed octopamine receptor subtype in their RNA-seq dataset and therefore the physiological effects of epinastine are most likely mediated through this receptor. However, I do not find this argument fully convincing.

First, relatively low transcript abundance of other OA receptor subtypes does not exclude their physiological contribution. Even receptors expressed at lower levels may play important functional roles, particularly in specific neuronal populations or flight-related tissues. Therefore, the possibility that epinastine affects multiple OA receptor subtypes cannot be excluded.

Second, although epinastine is widely used as a pharmacological tool to inhibit octopamine signaling, its receptor pharmacology has not been comprehensively characterized. The study by Roeder et al. primarily employed radioligand binding assays, which provide information on receptor affinity but not on functional antagonism or subtype selectivity. Without systematic functional characterization across the insect octopamine receptor family, it remains difficult to exclude contributions from other OA receptor subtypes or potential off-target effects.

A more convincing pharmacological strategy would be to demonstrate similar results using an additional chemically distinct octopamine receptor antagonist. Concordant phenotypes obtained with two independent antagonists would substantially strengthen the conclusion and reduce concerns regarding off-target effects.

(2) The OA supplementation experiments should be interpreted more cautiously.

The authors correctly acknowledge that exogenous octopamine produces only transient elevations in signaling. However, I do not find the comparison with synthetic agonists entirely appropriate.

Although synthetic agonists such as amitraz generally produce more prolonged receptor activation than endogenous octopamine, the more fundamental difference lies in their physicochemical properties. Octopamine is a highly polar endogenous amine that exhibits limited tissue penetration and is rapidly cleared through uptake and metabolic pathways. Consequently, exogenously administered OA is unlikely to efficiently reach relevant target tissues or receptor populations in a manner comparable to endogenous neurotransmitter release. In contrast, the greater lipophilicity of amitraz facilitates its distribution into target organs and enables more sustained receptor engagement following systemic administration.

More importantly, the observation that OA supplementation partially rescues flight behavior does NOT necessarily establish that altered endogenous OA signaling is the primary mechanism underlying the virus-induced phenotypes. Such rescue experiments demonstrate that pharmacological enhancement of octopaminergic signaling can modulate the phenotype, but they do NOT provide direct evidence that endogenous OA levels or OA signaling are altered by viral infection. Therefore, these experiments should be interpreted as supportive rather than mechanistic evidence.

(3) Direct quantification of octopamine remains the major missing evidence.

The authors acknowledge that direct measurements of octopamine and tyramine would strengthen their conclusions but argue that technical limitations and cost prevented these analyses. While these practical considerations are understandable, they do not compensate for the absence of the critical mechanistic evidence.

Overall, I appreciate the authors' revisions and agree that the manuscript provides interesting evidence that octopaminergic signaling is associated with virus-dependent changes in honeybee flight performance. However, I do not believe that the current data are sufficient to support the stronger mechanistic claims regarding regulation of the OA pathway or the specific involvement of the AmOARβ2 receptor.

Unless direct measurements of endogenous OA (and ideally tyramine) can be provided, I recommend that the authors substantially moderate the mechanistic conclusions throughout the manuscript, including the Abstract, Results, and Discussion. The study should be presented primarily as evidence for a pharmacological association with octopaminergic signaling rather than as definitive proof of the proposed mechanistic model.

# Author comment: Author response

**DOI:** 10.7554/eLife.111072.3.sa2

**Authors:** Naomi G Kaku, Michelle L Flenniken

**Affiliations:** Montana State University Bozeman United States; Montana State University Bozeman United States

The following is the authors’ response to the original reviews.

**eLife Assessment:**

This important study investigates how distinct honey bee viruses differentially alter flight performance through interactions with octopamine signaling pathways. The combination of behavioral flight assays, pharmacological perturbation, and transcriptomic analyses provides solid evidence that virus-specific effects on flight are associated with octopamine signaling. However, some of the stronger mechanistic conclusions regarding direct regulation of octopamine signaling remain incomplete without more specific validation of receptor-level effects and direct quantification of octopamine levels or signaling activity.

We revised some of text in the manuscript, since we agree that octopamine and tyramine quantification would strengthen the mechanistic interpretation of our findings. While we acknowledge that direct measurements of OA and tyramine would provide valuable complementary evidence, the current study relies on multiple independent lines of evidence—including gene expression analyses, OA supplementation experiments, and behavioral measurements—that collectively support a role for octopaminergic signaling in mediating the observed effects. The revised text better reflects the data included in this paper.

**Public Reviews:**

**Reviewer #1 (Public review):**

Summary:

Kaku and Flenniken investigate the mechanistic pathways through which specific viral infections alter the flight capabilities of honey bees. Building on their previous discovery that DWV impairs flight while SBV unexpectedly enhances it, the authors hypothesized that these behavioral shifts are driven by interactions with the insect's octopamine (OA) signaling pathway, which is responsible for the "fight-or-flight" neurohormonal stress response and energy mobilization. To test this, the authors experimentally infected adult honey bees with DWV or SBV and pharmacologically manipulated the OA pathway using either octopamine supplementation or epinastine (EP), an OA-receptor antagonist. They then evaluated the bees' flight performance (distance, duration, and speed) on custom flight mills and profiled their gene expression using qPCR and RNA sequencing.

Strengths:

A major strength of this study is the high prevalence of preexisting background DWV and SBV infections in the honey bee cohorts, which meant there were no completely "virus-free" control groups. However, the authors successfully mitigated this limitation by rigorously quantifying viral RNA copies for every individual bee via qPCR and utilizing these viral abundances as continuous variables in powerful linear mixed-effect models.

Weaknesses:

The primary weakness lies in the methodology used for targeted pharmacological manipulations, as well as the lack of OA quantification across different treatments. Thus, their claims are not sufficiently supported by the current data.

We thank Reviewer #1 for these comments.

(1) The authors utilize Epinastine to block octopamine signaling, describing it as a highly specific OA receptor antagonist. However, pharmacological inhibitors often lack absolute specificity. Epinastine might bind to other octopamine receptor subtypes present in honey bee neural and flight muscle tissues, or it could potentially cross-react with tyramine and dopamine receptors. Without further genetic validation (e.g., RNA interference targeting specific receptors), it is difficult to definitively conclude that the altered flight performance is solely due to the blockade of the specific Oβ−2R pathway.

We thank the reviewer for this thoughtful comment and agree that pharmacological approaches have inherent limitations with respect to receptor specificity. However, among the available octopamine receptor antagonists, epinastine is considered one of the most selective compounds for insect octopamine receptors. Roeder et al. (1998) reported that epinastine exhibits affinities for octopamine receptors that are at least four orders of magnitude greater than those for other insect biogenic amine receptors, including dopamine, tyramine, histamine, and serotonin receptors. We updated the text to include this information.

Honey bees encode four β-adrenergic-like receptors (AmOARβ1- AmOARβ4) and one αadrenergic-like receptor (AmOARα1). Our transcriptomic analyses indicated that expression of AmOARβ2 was substantially higher than that of other octopamine receptor genes. Specifically, AmOARβ4 transcripts were not detected in our RNA-seq datasets, while AmOARβ1 and AmOARβ3 were expressed at very low levels in most samples (Supplementary Table S9; Figure S5). Although AmOARα1 transcripts were detected in some samples, expression levels were consistently lower than those of AmOARβ2. These observations support the interpretation that the physiological effects observed following epinastine treatment are primarily mediated through disruption of AmOARβ2 signaling. We updated the text to include this information.

We agree that receptor-specific genetic approaches would provide valuable complementary evidence. RNAi-mediated knockdown of AmOARβ2 is an attractive future direction; however, RNAi efficacy in honey bees is variable and influenced by factors including transcript turnover rates. In addition, dsRNA treatments can induce sequence-independent antiviral effects that could confound interpretation in studies involving viral infection (Flenniken and Andino PONE 2013; Brutscher, Daughenbaugh, and Flenniken Sci Reports 2017). We have revised the manuscript to more explicitly acknowledge these limitations and to clarify the basis for our interpretation of the epinastine experiments.

(2) As a natural neurotransmitter, insects have evolved highly efficient "cleanup" mechanisms. OA is rapidly cleared from the synaptic cleft via reuptake transporters and quickly inactivated by enzymes such as N-acetyltransferase (NAT) or Monoamine Oxidase (MAO). Consequently, an injection of OA produces only a transient "pulse" of activity. It is often a poor "tool" for inducing prolonged physiological effects compared to synthetic formamidines like Amitraz.

We thank the reviewer for this important point regarding the pharmacokinetics of octopamine. We agree that octopamine is rapidly metabolised and cleared under physiological conditions and that exogenous administration is unlikely to precisely mimic endogenous signaling dynamics. Our goal was not to induce a prolonged pharmacological activation of octopamine signaling comparable to that produced by synthetic agonists such as amitraz, but rather to determine whether increasing octopaminergic signaling could mitigate the flight impairments associated with DWV infection. Octopamine was administered either by injection or through feeding (Lines 86-89), both of which resulted in significant improvements in flight performance in DWV-infected bees (Figure 2). The observation that two independent delivery methods produced similar outcomes supports the conclusion that enhanced octopaminergic signaling can partially rescue the DWV-associated flight phenotype. We have revised the manuscript to clarify this distinction and to acknowledge that exogenous octopamine administration likely produces transient elevations in signaling rather than sustained receptor activation.

(3) The study relies heavily on transcriptomics and quantitative PCR to measure the mRNA expression of key synthesizing enzymes, namely tyrosine decarboxylase (tdc) and tyramine βhydroxylase (tβh), to infer the activation or suppression of the octopamine pathway. However, changes in enzyme synthesis at the RNA level are often insufficient to accurately reflect the true physiological levels of biogenic amines. To robustly prove the authors' hypothesis of a "feedback loop that regulates intracellular OA concentrations", direct quantification of actual octopamine and tyramine titers in the bees (e.g., using high-performance liquid chromatography or mass spectrometry) is necessary.

We thank the reviewer for this comment and agree that octopamine and tyramine quantification would strengthen the mechanistic interpretation of our findings. Previous studies have successfully quantified OA in honey bees using HPLC-based approaches, including KayaZee et al. (2022, eLife), who measured OA in honey bee muscle tissue (both naturally occurring levels and levels post-treatment with 10 mM OA), and Cook et al. (2017, J. Exp. Bio) who quantified OA in pooled honey bee brain samples.

Prior to submission, we inquired with our institutional mass spectrometry facility regarding the feasibility of measuring OA in individual honey bee samples. The expected concentrations of OA in our samples was below their limit of detection, so we did not pursue these analyses at that time. During the review process, we explored the possibility of analyzing a subset of samples at external facilities that may have the sensitivity required to quantify OA and tyramine in honey bee tissues. Since such analyses would require substantial resources, with estimated costs of approximately $5,000–10,000 for 12–15 samples that have been stored in the -80C since the study, rather than flash-frozen in liquid nitrogen as described by Zee et al. 2022. While we acknowledge that direct measurements of OA and tyramine would provide valuable complementary evidence, the current study relies on multiple independent lines of evidence— including gene expression analyses, OA supplementation experiments, and behavioral measurements—that collectively support a role for octopaminergic signaling in mediating the observed effects. We thank the reviewer for this valuable suggestion. While these analyses are beyond the scope of this study, we will consider using this approach in future studies.

**Reviewer #2 (Public review):**

Summary:

This highly original and well-designed study provides insight into how honeybee picorna-like viruses, Deformed wing virus (DWV) and Sacbrood virus (SBV), affect flight performance, and reveals the role of the octopamine (OA) pathway in virus-honeybee interactions. The authors used a flight mill to quantify the flight performance of bees with different levels of DWV and SBV. Bees were treated with OA and/or epinastine (EP) - an OA receptor antagonist; the study also quantified virus loads and expression of two key genes involved in OA biosynthesis.

The results showed that reduced flight performance associated with high DWV levels could be alleviated by OA administration. In contrast, increased levels of SBV had the opposite effect, leading to enhanced flight performance. This suggests distinct physiological responses to DWV and SBV infections. Administration of EP had led to a reduction of flight performance in SBVinfected bees, indicating the involvement of the OA pathway.

The authors also quantified levels of mRNAs of enzymes involved in OA synthesis, tyrosine decarboxylase (TDC) and tyramine beta-hydroxylase (TbH), and concluded that DWV induced expression of TbH, while SBV upregulated expression of TDC. Furthermore, the study identified upregulated and downregulated genes in response to SBV, DWV and DWV in combination with OA.

Strengths:

The study reported opposing effects of infections of related viruses, SBV and DWV, on honeybee flight performance, and identified the central role of the octopamine (OA) signaling pathway in the effect of viruses on honeybee flights.

These findings were achieved by using a combination of approaches, including experimental measurement of flight distance, virus infections, and introduction of OA and EP. Experimental work with honeybees is technically challenging and requires specialized expertise, which makes the results produced in this study more valuable.

DWV and SBV are among the most important honeybee pathogens affecting honeybee health and threatening the pollination service. Therefore, an understanding of the mechanisms underlying DWV and SBV pathogenesis has the potential to develop novel approaches to mitigate the negative impact of these viruses.

Weaknesses:

No weaknesses were identified by this reviewer.

We thank Reviewer #2 for these comments

**Recommendations for the authors:**

**Reviewer #2 (Recommendations for the authors):**

I have only minor suggestions for the manuscript.

(1) L. 45-46

Please note that not only high virus levels have a negative impact on honeybees. Low levels of DWV, typical of covert infections, can have long-term deleterious effects on honeybee foraging and survival. Please include citation (e.g., Benaets et al, 2017, Proc Biol Sci (2017) 284 (1848): 20162149. https://doi.org/10.1098/rspb.2016.2149).

We thank the reviewer for these comments and edited the text accordingly, and apologize for our inadvertent omission of Benaets et al 2017, which is cited in our previous publication.

(2) L. 113

Clarify what is meant by "high DWV levels"

"i.e., 10^8 copies / 2 ug RNA" -> "i.e., above 10^8 copies / 2 ug RNA"

We thank the reviewer for this comment and corrected this in the text.

(3) L.115

"..mock infected bees.." /Figure 2A.

Did these bees have low levels of DWV, below 10^8 / 2 mg RNA? What was the level of DWV in these bees?

Mock-infected bees had an average of 3x10^5^ DWV copies and 2x10^3^ SBV copies per 2 µg RNA (reported in Lines 107-109 in revised manuscript, a few lines before in original manuscript).

(4) Figure 2 / Legends to Figure 2

Note that in Figure 2 legends, the grey areas show 95% confidence intervals for regression lines.

We thank the reviewer for this comment and added this in the text.

(5) Figure 2 / Legends to Figure 2

Consider including correlation coefficients (R) and p-values for each of the regression lines in Figures 2A-F. (These could be included in the Figure 2 legends).

We thank the reviewer for this suggestion and agree that providing sufficient statistical information is important for data interpretation. Because the analyses presented in Figure 2 are based on linear mixed-effects models that incorporate both fixed and random effects, the statistical outputs are more complex than those associated with simple linear regressions. For figure clarity, we chose not to include all model statistics within the figure panels or legends and the key statistical results, including p-values and model fit metrics (R^2^ values), are reported in the main text (Lines 121+). In addition, complete model outputs, including all relevant coefficients, correlation estimates, and associated statistics, are provided in Supplemental Data Sheet S4. To address the Reviewer’s comments, we revised the figure caption to improve clarity and include key p-values.

We believe this approach better balances accessibility in the main figures with comprehensive reporting of the statistical analyses and thank the Reviewer for this useful suggestion.

(7) L.357-377 - virus-specific responses

A previous honeybee transcriptome analysis study, which showed different responses to DWV and SBV, could be cited (Ryabov E. 2016. PeerJ 4:e1591 https://doi.org/10.7717/peerj.1591).

We thank the reviewer for this point and included this citation in line 338 of original manuscript (line 349 in revised, tracked-changes manuscript).

(7) L. 412

"bees were collected 24 hours prior to eclosion" -> e.g. "bees were collected at pupal stage 24 hours prior to eclosion"?

Specify if dark-eyed pupae were collected to make sure eclosion in 24 hr.

We thank the reviewer for making this point, and we revised the methods and results text to improve clarity.
